# The health care sector in the economies of the European Union: an overview using an input–output framework

**DOI:** 10.1186/s12962-021-00258-8

**Published:** 2021-01-19

**Authors:** Pedro Gutiérrez-Hernández, Ignacio Abásolo-Alessón

**Affiliations:** Departamento de Economía Aplicada y Métodos Cuantitativos, Facultad de Economía, Empresa y Turismo, Universidad de La Laguna (ULL), San Cristóbal de La Laguna, Santa Cruz de Tenerife, Spain

**Keywords:** Health care sector, Supply and demand, Input–output analysis, Apparent labour productivity, European Union

## Abstract

**Background:**

This study aims to analyse the relative importance of the health care sector (health care activities and services), its interrelations with the rest of productive activities, aggregate supply and demand, employment requirements and apparent labour productivity in the European Union (EU) economy as a whole, and in the economies of member countries.

**Methods:**

The methodology used is based on input–output analysis. Data are extracted from National Accounts and, specifically, from the input–output framework for 2010. Data in national currencies are adjusted using as a conversion factor, specific purchasing power parities for health.

**Results:**

In the EU, market production predominates in the provision of health care activities, which are financed mainly by public funding. However, there is significant variability among countries, and, in fact, non-market production predominates in most EU countries. The health care sector has direct backward and forward linkages lower than the average for all sectors of the economy and the average for the services sector. Thus, this sector is relatively independent of the rest of the productive structure in the EU. The health care activities industry is key because of its ability to generate value added and employment. Regarding apparent labour productivity, there are significant differences among EU countries, showing that productivity is positively related to the weight of market production in health care activities and negatively related to the number of hours worked per person employed.

**Conclusions:**

Our results provide useful insights for health authorities in the EU, as they analyse the effect of health policies on macroeconomic indicators using an input–output framework, as well as comparing these effects with those in EU member countries. To the best of our knowledge, an analysis of the health care sector in the EU economy and the countries that integrate it using an input–output framework has not been undertaken. In addition, to compare health care expenditure between countries, data in national currencies have been adjusted using specific purchasing power parities for “health”, and not ones referring to the total economy (GDP), which is common practice in many previous studies.

## Background

The European Union does not have a unique health care system, rather, different countries have different health care systems that stem from different historical, political and socioeconomic traditions [1]. In general, each country has a public health care system that provides a wide range of health care services for free at the point of consumption to their populations. This is organised through a national health service (or Beveridge model) characterised by financing through general taxation and covering all residents (such as in Cyprus, Denmark, Finland, Ireland, Italy, Latvia, Malta, Portugal, Spain, Sweden, United Kingdom). Alternatively, a social security system (or Bismark model) is used and financed by compulsory fees paid by workers and employers that are allocated to funds for health care services independent of the government (as happens for the remaining seventeen EU-28 countries). As well as public funding, a non-negligible private funding share is present in most EU countries made up of co-payments, out-of-pocket payments, or private health insurance. Either way, all European health care systems share the common objectives of ensuring proper access to health care and responding efficiently to their population’s health care needs [2, 3].

In addition to the organisation system, it is interesting to compare across EU countries the roles of the health care sector within their economies. With this aim, the first key economic issue is to measure the amount of funding -public and/or private- devoted to the final consumption of health care goods and services, which, in turn, can be publicly and/or privately provided. A second important issue is to determine the interrelations of the health care sector with the rest of productive activities, as well as its contribution to value added and productivity in the economy. Regarding the former, this has been tackled in many related studies and publications employing databases or information from indicators extracted from the OECD (Health Statistics) and Eurostat (Health Care). The main methodological base on which this information is prepared is the Manual of the System of Health Accounts (SHA) [4], whose latest edition was published in 2011 and is coordinated by the OECD, Eurostat and the WHO. Although the SHA adopts concepts and definitions derived from the Systems of National Accounts (SNA) [5], there are important differences that must be considered when comparing results generated by these systems (SHA and SNA).^1^ For example, the range of activities or products considered as belonging to the health sector is wider in the SHA and current expenditure estimated is greater than that under the SNA. However, in this research, the SNA is used (input–output framework), because it is the only source of information that allows the second economic issue mentioned above to be tackled, i.e. the interrelations between the health care sector and the rest of the economy. This task cannot be undertaken with the information provided by the SHA.

Thus, the aim of this study is to analyse the productive structure of the health care sector in the European Union and the interrelations between the health care sector and the rest of the economy, as well as to measure value added (income), employment generated and other relevant economic indicators. This information is useful for EU health authorities as it facilitates the analysis of the effect of health policies on macroeconomic indicators using an input–output framework, as well as comparing these effects between different EU countries. To the best of our knowledge, a similar analysis has not been undertaken so far. The rest of the paper is organised as follows. In the second section, the research methods are presented. The third section describes the results obtained. A discussion of these main findings is presented in section four. The paper ends with the main conclusions.

## Methods

The methodology used in this work is based on an input–output analysis. By using an input–output framework, it has been possible to quantify the interrelations between the health care sector and the rest of the economy, as well as to measure the value added (income), employment generated and other relevant economic indicators.^2^

The input–output framework is made up of three types of tables: supply table, use table and symmetric input–output table (hereinafter SIOT).^3^

In supply and use tables, the information recorded in the columns is organized by industry. In this case, the information referring to the health care sector is collated in the industry called “health care activities”,^4^ which offers different products, such as, health care services (as principal activity^5^), or scientific research and development services, social work services^6^ or education services for training health personnel (as secondary activities^7^). Likewise, other industries, such as "social work activities", also produce health care services as a secondary activity.^8^

In the rows of the supply and use tables (also in the rows and columns of the SIOT), information is recorded by product. In the case of the health care sector, the information is presented by the product called “health care services”,^9^ which exclusively offers hospital services, medical and dental practice services and other health care services. In the same way, the product of social work services only produces this type of services, excluding, among other possible ones, those related to health care services.

As a result of the above, the output values recorded in the rows and columns of the supply and use tables for each industry and product may be different. These differences will be greater to the extent that an industry develops a greater number (and greater value added) of secondary activities. This situation is not presented in the SIOT as its rows and columns are organised product by product or industry by industry. In any case, and provided that the supply and use tables are valued at the same prices,^10^ for the whole economy, the total output by product or by industry must match each other.

The geographical scope of this study is the European Union and the countries that integrate it. Thus, the main data source used in this paper corresponds to the Input–Output Framework of different countries of the European Union (EU), as well as for the whole of the EU (28 countries) and the Economic and Monetary Union (EA, 19 countries), published by EUROSTAT in its database*.* The methodological basis that supports such data is the European System of National and Regional Accounts (ESA 2010). The reference year is 2010, the last year for which, during the preparation of this study, a significant number of tables derived from the input–output framework on the methodological basis of the European System of Accounts (hereinafter ESA 2010) [11] were available.^11^

To compare health care expenditure between countries, data in national currencies has been homogenized through purchasing power parities (PPPs). The results are presented in a common artificial currency in the European Union called the purchasing power standard (PPS) (see Eurostat [12] and Eurostat-OECD [13]). The PPPs used are those specific to the health component, and not the aggregates for GDP (as is common practice in other published studies [14, 15]. They correspond to a review carried out by Eurostat-OECD in December 2016, which, in addition to the update required by the new ESA 2010 [11], introduced other methodological developments, such as those related to the new calculation method for specific health PPPs, which incorporated a new approach that was based on outputs, and not on inputs, as had been happening until that time. In this regard, see Koechlin et al. [16].

In addition to the analysis of the composition of aggregate supply and demand for the health care sector in the European Union, we explore six economic issues of the health care sector that include: (1) The direct backward linkages of the health care activities industry and the direct forward linkages of health care services, as the main characteristics of the productive structure and the intersectoral relationships of the health care sector. (2) Simple output multipliers. (3) GVA requirements. (4) Employment requirements (hours worked). (5) Final consumption expenditure, distinguishing between general government, household, and total final consumption expenditure. (6) Approximation of apparent labour productivity.

Regarding the direct backward linkages of the health care activities industry, we must start by saying that, in the national accounts context, the cost structure of the production process in each industry or in the production of each type of product consists of two elements: (1) the costs of using different types of products (intermediate consumptions or inputs) and( 2) the costs of production factors (labour—represented by employee compensation- and capital—represented by gross operating surplus), which added together constitute the Gross Value Added (GVA hereinafter). Direct backward linkages measure the weight that costs related to the use of different products (i.e. intermediate consumptions or inputs) represent in the production (output) of a particular industry or in the production of a particular type of product. It can be understood as an indicator of the ability of a particular sector to stimulate (backward) the development of other sectors from which it acquires inputs that are necessary for its production process [17–19]. The analysis of direct backward linkages can be approached through two of the abovementioned tables: the use table, which provides information regarding the use of the different products (intermediate consumptions or inputs) in each industry and through the SIOT (at basic prices, product by product) that offers information regarding the use of different products in the production of each type of product. Given that in the EU and EA as a whole, the supply of secondary products by the health care activities industry is almost insignificant (less than 3% of the total supply of products of this industry), and provided that both tables are valued at the same prices (basic prices), the results obtained should not be very different.^12^

With respect to direct forward linkages of health care services, the rows of the use and SIOT tables present the two possible types of use for each type of product: (1) as intermediate consumption in the production of the different industries or products, and (2) to meet final uses (final demand) whether of final consumption, investment or exports. Therefore, direct forward linkages would quantify the weight that intermediate consumption represents in total possible uses (output). It can be understood as an indicator of the ability of a particular sector to promote (forward) the development of other sectors that need such inputs for their corresponding production processes. Since the use table at basic prices is a product by industry table, the information contained in its rows in the table for final demand must coincide with that in the same table of the SIOT (product by product), provided that both tables contain no errors.^13^

The simple output multipliers can be calculated in two ways. From the supply and use tables, they are obtained as the sum of the elements of each column of the corresponding matrix [(I − DZ) − 1 D].^14^ In the specific case of the matrix column corresponding to the industry of health care activities, this sum would indicate the total production (direct and indirect)^15^ of all industries of economy (including health care activities) per unit of additional final demand for health care services (assuming stable or constant coefficients of matrices Z and D). From the SIOT matrix, the elements of each column of the corresponding matrix should be summed (in this case, the Leontief inverse matrix [(I − A)^−1^]). In the specific case of the column of the inverse matrix corresponding to health care services, this sum would indicate the production of all products (including health care services) per unit of additional final demand for health care services (assuming stable or constant coefficients of matrix A).

With respect to the GVA requirements, by combining the information on GVA at basic prices (use table) and production at basic prices (supply or use table), it is possible to calculate the GVAbp (basic prices) that is generated directly in each industry per unit of output (as a quotient between GVAbp and production at basic prices). This information does not coincide exactly with that obtained from the SIOT, since in this table the GVAbp provided is the one generated by the production of each type of product and not in each industry.

Another economic issue is related to employment requirements (hours worked). Information on the production at basic prices of each industry (tables of supply or use) is combined with that relating to hours worked^16^ (as an indicator of employment) in each industry, which is obtained from the employment data by industry from Eurostat. It is then possible to calculate the required hours worked in each industry to obtain a unit of output (as a quotient between the number of hours worked and production at basic prices).^17^

Final consumption expenditure (FCE) is also part of this analysis. Regarding General Government (GG), the FCE of the GG (current expenditure^18^) consists of two components: (1) costs incurred in the provision of non-market services (mainly employees’ compensation, intermediate consumption and fixed capital consumption), from which payments at economically non-significant prices made by households for the use of such products would be deducted, and which, for the most part,^19^ would constitute “non-market social transfers in kind” (hereinafter, non-market STIK); (2) acquisitions by the GG of products from market producers, which are supplied to households without any transformation (deducting any possible co-payments made by households), in the form of “social transfers in kind provided by market producers” (hereinafter, market STIK). Specifically, the FCE of the GG arising from health care services (rows of the use table or the symmetric table by products) is also composed of the same two components already mentioned. Although it is limited to the provision of health care services, both in terms of production costs and in relation to market STIK, which are limited, basically, to the contracting out of heath care services excluding, among others, expenditure on “extra-hospital”^20^ pharmaceutical products. Therefore, GG’s FCE would exclusively comprise services of an individual nature (health care), since those of a collective type would be registered (use table or SIOT by products) in the rows with the corresponding products equivalent to the mentioned collective services. With respect to households, household FCE^21^in health care services (rows of the use table or SIOT by products) includes the expenditure made in market health care services plus possible payments (at economically non-significant prices) for non-market health care services. Finally, the sum of both (GG and households) makes up total final consumption expenditure.

A final economic issue of interest is that of apparent labour productivity. This measures the relationship between the production obtained and the amount of labour incorporated into the production process (production divided by labour incorporated). For the first variable, we use both the GVA at basic prices (approximation to the income generated) and output, both data taken from the use tables at basic prices. For the second, we use the number of hours worked.^22^ Apparent labour productivity indicates apparent level of efficiency of the use of labour. The term apparent refers to the fact that productivity depends on all production factors and the way in which they are combined.

As mentioned previously, the specific PPPs for health care published by Eurostat-OECD, incorporate the new approach based on output (for example, cost of a heart transplant), and not on inputs (e.g., hourly wage of a nurse), which has been the method traditionally applied in comparisons of PPPs for non-market production between countries. However, this method ignores differences in productivity between countries. As Schreyer (page 116) [21] points out, the specific PPPs for health based on output record price ratios (unit costs) for health services in different countries, which when applied to the monetary values of production costs or consumption expenditure on health allows a comparison in volume of such services between the countries considered, and, in principle, shows the differences in productivity between countries. “In a non-market context, even when the money value of output is measured by the monetary value of inputs, it is possible to derive the right volume indices of output as long as the estimated PPPs are based on cost measures per unit of outputs, and not on cost measures per unit of inputs” (Schreyer, page 130 [21]). The new health-specific PPPs published by Eurostat-OECD [13] represent an approximation to this objective, for which “quasi-prices” (negotiated or administrative prices or tariffs) are used to calculate the value of the production of these services, and not the prices of the different inputs. It should also be taken into account, that the new PPPs used here do not yet incorporate the possible differences in quality of the provision of such services between countries, which is a limitation. On all these issues, see also Koechlin, F. et al. [16, 22, 23], Schreyer [24] or Lorenzoni, L. and Koechlin, F [25].

## Results

### Productive structure and intersectoral relations of health care activities and services

#### Direct backward linkages of the health care activities industry

**Fig. 1:**
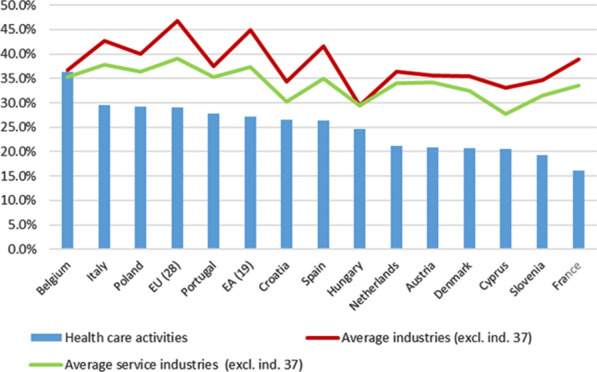
Health Care Activities. Direct backward linkages (%) (domestic at basic prices). 2010. *Source:* Prepared by authors with Eurostat data, "Use table at basic prices (domestic)"

We will begin with an analysis of the direct backward linkages with domestic supply (use table), which are relevant to evaluate the degree of interrelation with other productive activities in the territory. In the whole of the EU (Fig. 1), the acquisition of domestic intermediate inputs (products at basic prices) represents 29.0% per unit of production of health care activities (industry No. 33), when the average^23^ for the 36 industries considered represents 46.7% (27.2% and 44.8%, respectively, in the whole of the EA). These linkages of the health care activity industries are also lower than those registered for the average of all services industries (39.0% in the EU and 37.4% in the EA). For the industry of health care activities, the countries that are above the EU average are, in this order, Belgium, Italy, Poland and Portugal. In particular, the case of Belgium stands out, in which the weight of the intermediate inputs of products on the production value of health care activities is very similar to that of the average for all industries: 36.3% compared to 36.7% recorded for the average of the 36 industries. This greater backward linkage is very concentrated in the health care sector, itself, since the acquisition of health care services by health care activities industry (“intra-consumption”)^24^ represents 13.4% of the production value, a weight much higher than that registered in any of the countries analysed, and in the EU and EA as a whole (4.9% and 4.5%, respectively).

The detail by type of products together with those related to each of the countries analysed are presented in Table S5^25^ of Additional file 4. On the other hand, in Table S7 of Additional file 4, the weights for each type of product are presented (as % of the total of intermediate inputs of domestic origin used by health care activities).

The structure of the weights of each type of product in total intermediate consumption of health care activities shows, with slight variations between the countries analysed, that the main “costs” are concentrated in four types of products: basic pharmaceutical products and their preparation (No. 8, which only includes those for in-hospital use, which represents 9.8% in the EU as a whole; 8.8% in the EA), wholesale and retail trade services (No. 19, 13.9% and 15.7%, respectively), health care services (No. 33, "intra-consumption", 16.9% and 16.4%) and administrative and support services "(No. 30, which includes, among others, rental services—non-real estate—and leasing, employment-related services, travel agency services, security, cleaning and other business assistance services, 7.6% and 8.1%). In the EU and EA, the joint use of these four types of products represents approximately 50% of total intermediate inputs used by health care activities. Other products that also represent significant costs, although less important than the previous ones, are those related to “furniture, other manufactured products and repair and installation services of machinery and equipment” (No. 15), “other business services” (No. 27), such as legal, accounting and consulting services, technical analysis and testing services, and “real estate services” (No. 26).

If total (domestic and imports,^26^ basic prices) intermediate inputs used by the health care activities are now considered, it can be seen that in the EU as a whole, they represent 32.2% per unit of production, when the average for the 37 industries is 51.8% (30.2% and 51.5%, respectively, in the EA). The weight structure of each type of product does not show many differences from the previous one, so main costs remain the same.^27^ Perhaps, the most notable difference is the higher weight registered for the consumption of basic pharmaceutical products and their preparation, which in the whole of the EU and EA reaches 13.1% and 12.4%, respectively, indicating the importance of the external pharmaceutical market for Europe as a whole.^28^ In addition, the weight increases, although to a lesser degree, regarding the use of “furniture, other manufactured products and repair and installation services of machinery and equipment”, which ranges from 4.6 to 5.5% in the EU as a whole (from 4.9 to 6.6% in the EA).

If total (domestic and imported) intermediate inputs at purchasers’ prices are analysed, the weight structure of different products shows a major change, because of the different valuation systems. The reason is that purchasers’ prices imply that the distribution margins (trade and transport, mainly) are incorporated into the prices of the different products, to which the corresponding taxes on products are also added. This means that wholesale and retail trade services disappear as one of the main “costs” of health care activities, which, logically, is reflected in an increase in the weight of the other products used. There are no use tables available at purchasers’ prices for the EU and EA as a whole, but, for example, in most of the countries analysed, the weight of basic pharmaceutical products and their preparations on the total intermediate consumption increases by 4 or 5 percentage points compared to the use of these products at basic prices, with Spain standing out with an increase of more than 7 points (from 16.6 to 23.9%) Data on purchasers’ prices on the weights for each type of product for each of the countries analysed are presented in Table S9 of Additional file 4.

Therefore, we can affirm that the health care activities industry, regardless of the origin of the products used (only domestic or including imports) is characterized by presenting direct backward linkages below the average for all industries. The low use of intermediate inputs necessary for health care activities production contrasts with the relevant weights recorded for the primary inputs (value added, fundamentally), a general characteristic present in practically all industries producing services, although in health care activities, this low weight registers a greater intensity.^29^

#### Direct forward linkages of health care services

The main use of the production of health care services (domestic supply, at basic prices) is the final demand, and especially its component of final consumption expenditure (FCE), which represents approximately 93% of the total use in the EU and EA (with no excessive variations among countries). These percentages hardly vary in cases where the total outputs (domestic and imported origin) are considered or when the table is valued at purchasers’ prices.^30^

**Fig. 2:**
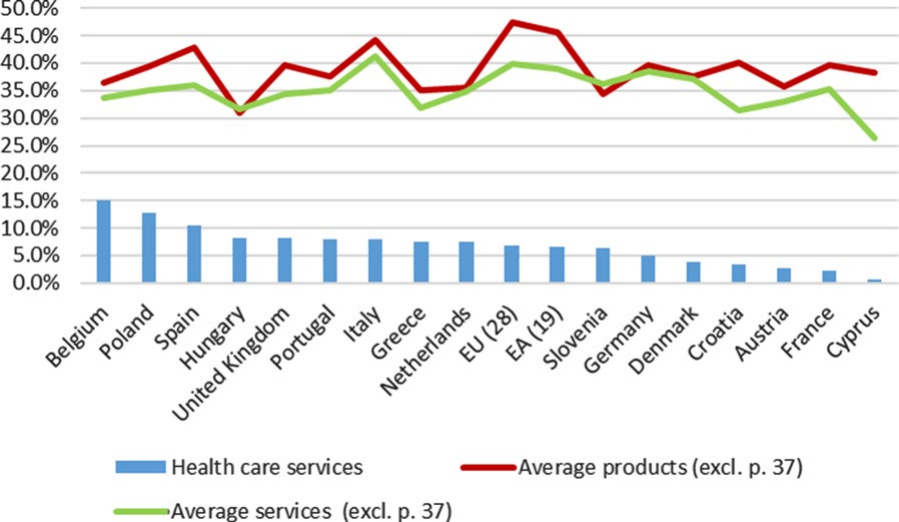
Health care services. Direct forward linkages (%) (basic prices, domestic). 2010. *Source:* Prepared by authors with Eurostat data, "Use table at basic prices (domestic)"/“Symmetric input–output table at basic prices (product by product) (domestic)"

The use of health care services, as individual services,^31^ is mainly oriented to final demand, and, fundamentally to the FCE. This implies that the use of such services by producing industries has a rather residual character (occupational health care, for example): for the EU and EA as a whole (Fig. 2), the total intermediate outputs (domestic, at basic prices) only represent 6.9% and 6.6%, respectively, of the domestic supply of health care services.^32^ These weights are significantly lower than the averages for the total products considered (46.2% in the EU and 44.4% in the EA), as well as the averages of such direct forward linkages for services (37.9% and 36.8%, respectively).^33^ However, there are some cases in which these weights are somewhat higher, such as Belgium (15.1%), Poland (12.7%) or Spain (10.5%), mainly as a result of the aforementioned "intra-consumption" of such services (14.3%, 8.7% and 5.6%, respectively, of the total output of such services).

In other words, the production of health care services is characterized by presenting forward linkages below the average of all products: their products are very little used in industry production.

#### Simple output multipliers

The simple output multipliers^34^ of the health care activities industry (the total production—direct and indirect—of all industries of the economy per unit of additional final demand for health care services) record values that are below the average multiplier for the 37 industries for all countries: in the EU (Table 1), the value of this multiplier is 1.53, while the average for all industries analysed is 1.90. In the case of the EA, these multipliers register the values of 1.48 and 1.84, respectively. If the comparison is made with the average multiplier of service industries, again health care activities has lower values, with the only exception of Belgium.

**Table 1:** Simple output multipliers. 2010

	Health care activities. Simple Output Multipliers (supply and use tables)					Health care services. Simple Output Multipliers (symmetric input–output table)				
	Health care activities	Average industries	Average industries (excl. ind. 37)	Average service industries	Average service industries (excl. ind. 37)	Health care services	Average products	Average products (excl. p. 37)	Average services	Average services (excl. p. 37)
EU (28)	1.528	1.899	1.923	1.703	1.741	1.528	1.899	1.923	1.702	1.740
EA (19)	1.480	1.838	1.861	1.656	1.692	1.480	1.838	1.861	1.655	1.692
Belgium	1.566	1.576	1.592	1.532	1.562	1.585	1.578	1.595	1.545	1.576
Denmark	1.309	1.547	1.556	1.488	1.501	:	:	:	:	:
Germany	:	:	:	:	:	1.404	1.654	1.672	1.573	1.605
Spain	1.450	1.752	1.773	1.593	1.626	1.443	1.760	1.781	1.572	1.603
France	1.269	1.661	1.679	1.544	1.575	1.269	1.661	1.679	1.545	1.575
Croatia	1.403	1.537	1.539	1.473	1.474	:	:	:	:	:
Italy	1.505	1.771	1.792	1.652	1.688	1.504	1.769	1.790	1.650	1.686
Cyprus	1.327	1.505	1.519	1.428	1.452	:	:	:	:	:
Hungary	1.354	1.421	1.433	1.411	1.434	1.351	1.428	1.440	1.411	1.434
Netherlands	1.330	1.561	1.576	1.501	1.529	:	:	:	:	:
Austria	1.338	1.587	1.603	1.534	1.564	1.336	1.596	1.613	1.531	1.561
Poland	1.484	1.668	1.686	1.584	1.616	:	:	:	:	:
Portugal	1.460	1.624	1.641	1.556	1.586	:	:	:	:	:
Slovenia	1.298	1.549	1.565	1.489	1.516	:	:	:	:	:
Greece	:	:	:	:	:	1.304	1.547	1.562	1.448	1.473
UK	:	:	:	:	:	1.414	1.599	1.616	1.526	1.555

^a^(:) not available

*Source:* Prepared by authors with Eurostat data, "Use table at basic prices (domestic)”/"Supply table at basic prices"/”Symmetric input–output table at basic prices (product by product) (domestic)”

The relatively low value of the aforementioned multipliers shows the weakness of the indirect effects arising in the productive structure as a result of changes in the final demand for health care services. Table 2 shows the values of these indirect effects, calculated from the information recorded in the SIOT (basic prices, domestic origin) of the different countries. In most countries for which the corresponding information is available, the indirect impact generated is substantially less than that recorded for the average of all types of products (slightly less than 50%), and lower than the average for the services. The exceptions are Belgium and Hungary, in which the indirect effect is barely inferior to all products average.

**Table 2:** Indirect requirements per unit of additional final demand. 2010

Indirect requirements^a^ on gross output in the whole economy per unit of additional final demand for each type of product (SIOT)											
	EU 28	EA 19	Spain	Germany	France	Italy	Belgium	Austria	Greece	Hungary	UK
Health care services	0.2379	0.2084	0.1813	0.1582	0.1082	0.2088	0.2121	0.1293	0.1038	0.1070	0.1507
Average products	0.4431	0.4010	0.3515	0.2717	0.2847	0.3486	0.2234	0.2524	0.1876	0.1420	0.2410
Average products (excl. p. 37)	0.4552	0.4121	0.3613	0.2792	0.2926	0.3583	0.2297	0.2595	0.1928	0.1460	0.2477
Average services	0.3292	0.2984	0.2454	0.2340	0.2288	0.2858	0.2119	0.2191	0.1393	0.1332	0.2020
Average services (excl. p. 37)	0.3471	0.3149	0.2590	0.2471	0.2415	0.3016	0.2236	0.2313	0.1470	0.1406	0.2132

^a^Indirect requirements have been calculated as the difference between the Leontief inverse matrix (symmetric input–output table) and the technical coefficients matrix (basic prices, domestic), also discounting the unit increase in production caused by the variation in corresponding final demand

**Source**: Prepared by authors with Eurostat data, “Symmetric input–output table at basic prices (product by product) (domestic)"

It should be noted that these indirect effects caused by the final demand for health care services now have a significant impact on other products, in addition to those already mentioned when direct backward linkages were analysed, and are the following: electricity, gas, steam and air conditioning (No. 16; 0.012 in the EU as a whole, 0.011 in the EA), transport and storage services (No. 20; 0.02 and 0.017, respectively), and financial services and insurance (No.25; 0.018 and 0.017).^35^

#### GVA requirements

**Fig. 3:**
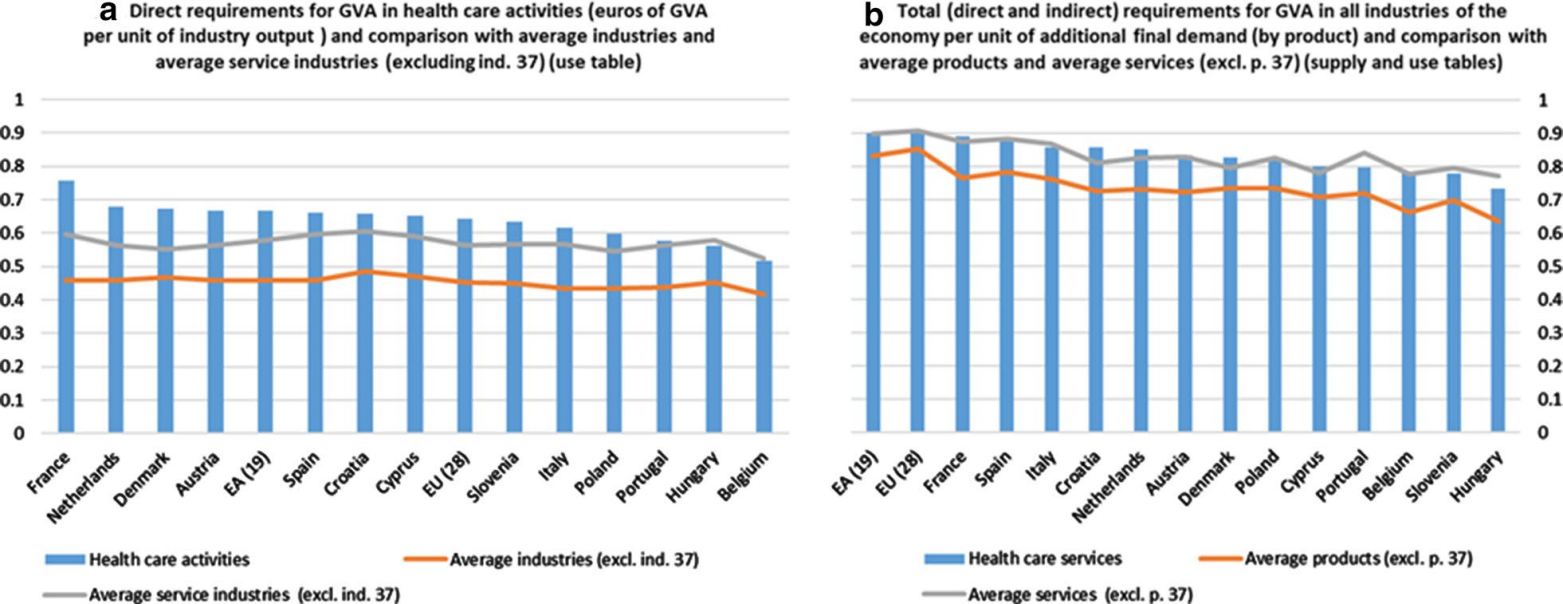
GVA requirements. 2010. *Source:* Prepared by authors with Eurostat data, "Use table at basic prices (domestic)"/"Supply table at basic prices"

The results (euros of value added per unit of industry output) are presented on the left side of Fig. 3a.^36^

For all the countries analysed, the GVA generated directly per unit of production in the health care activities industry is higher than the average for this indicator for the 36 industries considered. In the EU and EA, it has a value of 0.64 and 0.67, respectively, when the average values ​​for all industries are 0.45 and 0.46, respectively.^37^ Also, with the only exceptions of Belgium and Hungary, the indicator of this industry exceeds the average value of this indicator for all service industries.

The use of the direct coefficients of GVA in combination with the corresponding matrices, obtained from the supply and use tables or from the SIOT, allow total GVA generated in the economy (all industries, including health care activities) to be calculated (direct and indirect). In the case of the health care sector, supply and use tables provide the GVA (income) generated in all industries when the final demand for health care services increases by one unit (assuming stable or constant coefficients of the matrices Z and D). If the information contained in the SIOT were used for the calculation of the aforementioned requirements, the interpretation of the results would be similar to that indicated, although, as already mentioned, the GVAbp provided in this table is the one generated by the production of each type of product, not in each industry.

Using the matrices obtained from the supply and use tables (Fig. 3b), it can be seen that the final demand for health care services has the capacity to generate in the economy more value added (income) than the average of the 37 types of products (the same happens if product 37 is eliminated). The results of the comparison with the average of services depend on whether product 37 is included, and, in both cases, the results for the different countries analysed are not of the same sign. Thus, in the EU and EA, total GVA generated in all 37 industries when the final demand for health care services increases by one unit is 0.90, while the average for the 37 types of products records values of 0.86 and 0.84, respectively.

If the information contained in the SIOT (at basic prices, domestic supply) was used to calculate the requirements of GVA, the results obtained would indicate the following: final demand of health care services has the capacity to generate in the economy (in the production of all products) more value added than the average of the 37 types of products (the same happens if product 37 is eliminated). The only exception is in the United Kingdom, in which its indicator for health care services is somewhat lower than the abovementioned average (0.75 versus 0.76; same value in the case where product 37 is eliminated). In the EU and EA, the total GVA (direct and indirect) generated in the production of all products when the final demand for health care services increases by one unit is 0.90. The average for the 37 products records values of 0.86 and 0.84, respectively. These data coincide with those obtained from the information contained in the supply and use tables.^38^

As already noted, when simple multipliers are analysed, the weakness of the indirect effects in the productive structure resulting from an alteration in the final demand for health care services leads to lower multiplier effects. This justifies, as can be seen from the comparison between the two sides of Fig. 3, why the differences between the values for the averages and the direct GVA are greater than when the averages are compared with the total GVA generated by the health care services.

#### Employment requirements (hours worked)

The results are presented in Table 3, which also provides information on the average for the 36 and for the 18 service industries.^39^ Table S13 of Additional file 4 provides the data referring to the averages for the total industries, without excluding industry 37, as well as the indicators obtained on the basis of the information contained in the SIOT for the countries in which this document is available, thus verifying that the differences with those calculated from the supply or use tables are minimal.

**Table 3:** Health care activities. Direct requirements for employment (hours worked) per unit of industry output (hours worked per thousand EUR of output). 2010

	Health care activities	Average industries (excl. ind. 37)	Average service industries (excl. ind. 37)
EU (28)	22.3	15.3	18.2
EA (19)	20.0	12.9	16.7
Belgium	13.5	9.0	12.2
Denmark	16.5	8.7	11.4
Germany	:	:	:
Spain	19.5	15.6	21.0
France	19.2	10.7	14.3
Croatia	69.6	45.9	49.5
Italy	17.4	13.2	16.3
Cyprus	24.6	25.1	25.6
Hungary	65.5	39.6	47.2
Netherlands	16.9	10.2	14.3
Austria	19.8	12.9	16.6
Poland	74.2	45.8	53.2
Portugal	32.2	26.5	31.7
Slovenia	32.5	24.8	28.0

^a^(:) not available

*Source:* Prepared by authors with Eurostat data, "Use table at basic prices (domestic)"/"National Accounts employment data by industry"

For almost all the countries analysed (except for Cyprus), the number of hours (employment) required directly per unit of production in the industry of health care activities is higher than the average for the 36 industries considered. Thus, in the EU and EA, the indicator for health care activities industry has a value of 22.3 and 20.0, respectively, when the average values for all industries are 15.3 and 12.9, respectively. The high requirements of hours worked in some countries (Croatia, Hungary or Poland) should be highlighted, which significantly increases the degree of divergence with respect to what was observed in the previous indicator regarding GVA between different countries. However, it is necessary to specify that, in this case, the comparison between countries is distorted by the fact that, when the output is valued in euros, the price differences for health care between countries have a significant impact on the value of this indicator.^40^ With the only exceptions of Spain and Cyprus, the indicator of this industry exceeds the average value of this indicator for all service industries. Therefore, in almost all the countries analysed, this industry could be considered as key because of its ability to directly generate (in the industry itself) employment in the economy.^41^

**Fig. 4:**
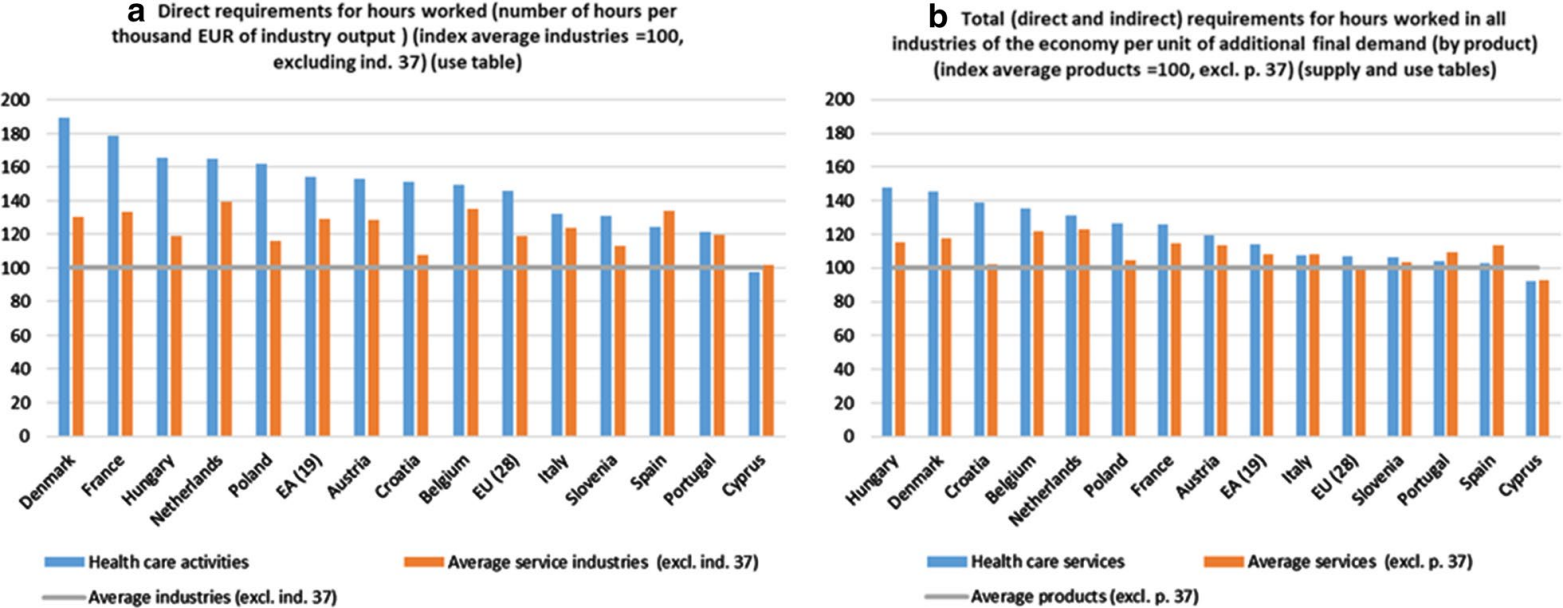
Requirements of hours worked (employment). 2010. *Source: *Prepared by authors with Eurostat data, "Use table at basic prices (domestic)"/"Supply table at basic prices"/"National Accounts employment data by industry"

Figure 4a presents the same information as in Table 3, but in the form of indexes of the indicator average for the 36 industries of each country. The countries in which the requirements of hours worked to produce health care activities exceed the average by the greatest amounts are Denmark, France, Hungary, the Netherlands and Poland.

The use of the indicator related to hours worked per unit of production in combination with the corresponding matrices, obtained from the supply and use tables or from the SIOT, allows the total number (direct and indirect) of required hours worked (employment) in the economy (in the production of all industries) to be calculated when final demand for health care services is increased by one unit (using the tables of supply and use).

The results obtained (Fig. 4b) show that in most of the countries analysed, final demand for health care services has the capacity to generate employment in the economy (all industries) with values above the average for the 37 types of products. This indicator registers a value of 30.4 for the EU and 26.5 for the EA, when the average for the 37 products is 30.0 and 25.3, respectively. However, this situation is not homogeneous for all the countries analysed. In Spain, Italy and Portugal the situation is as follows: 27.2 versus 28.0, 24.9 versus 25.9 and 44.7 versus 46.2, respectively.

When the total requirements are calculated based on the information contained in the SIOT (at basic prices, domestic supply), most of the countries analysed have values above the average of the 37 types of products. For the EU, this indicator records a value of 30.7 (26.8 for the whole of the EA), when the average is 29.9 and 25.1, respectively. However, this situation is not homogeneous for all the countries analysed. In Spain, Italy and Greece the situation is as follows: 27.4 versus 28.0, 25.1 versus 25.6 and 34.0 versus 43.3, respectively. Table S14 of Additional file 4 shows all the indicators used to prepare the previous figures, as well as those obtained from the information collected in the SIOT for those countries where such information is available.

Again, it can be asserted that, as a result of the weakness of the simple output multipliers, the differences between the values of the average and the direct and total requirements of hours worked are clearly reduced, which can be seen if we compare Fig. 4a, b.

### Aggregate supply and demand for the health care sector in the European Union

In the European Union, the output (domestic supply at basic prices) of health care activities represented 3.8% (917 billion euros) of the total output of all industries (3.8% in the Eurozone; 672 billion euros) in 2010, although several countries exceeded 4% (United Kingdom [5.1%], Ireland [4.7%], Greece [4.2%], and, Belgium, Portugal and Finland, with approximately 4%). By contrast, there were cases like those of Luxembourg, Latvia and Slovakia, in which this industry was under 2%.

**Fig. 5:**
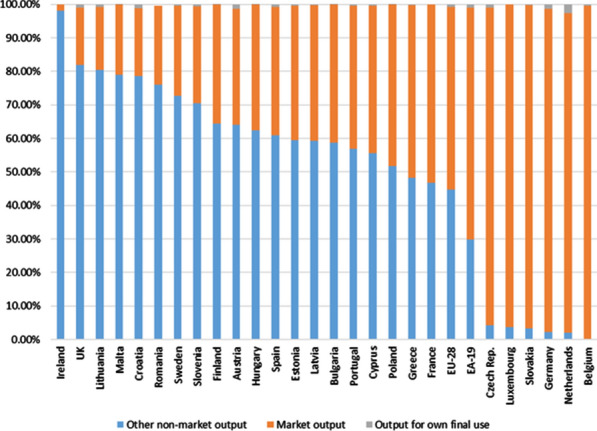
Health care activities. Types of output (% of total output). 2010. *Source: *Prepared by authors with Eurostat data, "Supply table at basic prices"

The information on health care activities collated in the supply table allows the supply to be disaggregated between market outputs: output produced for own final use and non-market output.^42^ Figure 5 presents this information for most EU member countries, as well as for the EU and EA combined.

As can be seen in the figure, output produced for own final use is totally residual in all cases.^43^

In most countries (18), the greatest weight corresponds to non-market output, while in eight countries, the main production is market output. Since non-market output can only be developed by non-market producers (mainly by the general government; hereinafter GG), it can be seen that in most EU countries the provision of health care services is public. However, at the aggregate level of the EU and the EA, the main production is that of the market (54.4% and 69.2%, respectively). However, it cannot be concluded that such producers are of a private nature, since this type of production can also be developed by non-market producers (although on a secondary basis—see footnote 7).

This perspective from the exclusive point of view of supply could lead to mistaken conclusions about the characteristics of health care systems. In cases where market output predominates, it could be mistakenly understood that its recipients (mainly households, given that these are individual services) must pay economically significant prices to be able to use or benefit from this supply. Therefore, this perspective must be complemented from a demand perspective (use).

**Fig. 6:**
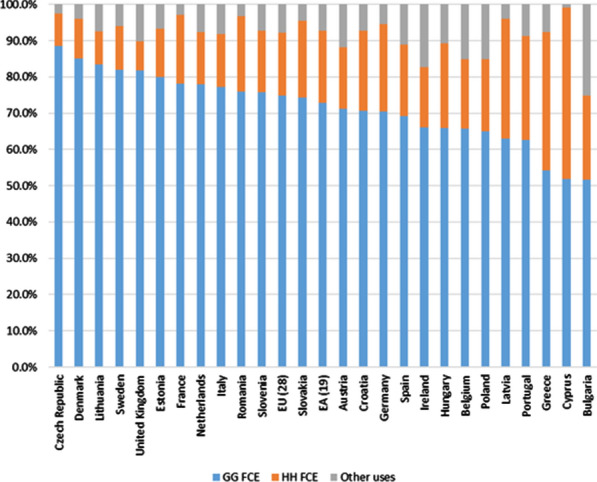
Health care services. Types of uses (% of total uses). 2010. *Note:* The “other uses” component includes all possible alternatives for the output of health care services, including intermediate outputs and other final uses (NPISH final consumption expenditure, gross capital formation (GCF) and exports). This use of GCF should not be interpreted as the investment expenditure made by health care services. The construction of the different input–output tables that are analysed only allows us to determine what the investment expenditure is by the set of industries of the economy, but not the expenditure carried out by each of the industries individually. *Source: *Prepared by authors with Eurostat data, "Use table at basic prices (domestic)"/“Symmetric input–output table at basic prices (product by product) (domestic)"

This information is provided by the use tables of countries (or also, if they exist, by SIOT ones), and a summary is presented in Fig. 6. For the EU, total demand (domestic supply at basic prices) of health care services was 3.8% (900 billion euros) of aggregate demand for all types of products (3.7% in the EA; 658 billion of euros). The countries that are above and below these aggregate weights basically coincide with those already mentioned for the supply of health care activities.

It has already been indicated above that the main use of health care service production (domestic supply, at basic prices) is final demand, and especially its component of final consumption expenditure (households, GG or NPISH). This represents approximately 93% of the total use of health care services supply in the EU and EA (with some variations among countries).

In all countries, household FCE is only 17.3% and 20.0% for the EU and EA, respectively, highlighting, instead, the main FCE, which is the GG (74.9% and 72.8% for the EU and EA, respectively). Even in countries where the market supply is very prominent (such as in Belgium (99.5%), Germany (96.4%) or the Netherlands (96.4%), household FCE as recipients of health care services is small (19.3%, 24.1% and 14.6%, respectively).

Thus, the main component of final demand is the FCE of the GG, despite the largest proportion of the supply corresponding to market production. This evidences the fact that the GG finances the provision of this market service by contracting out, allowing households to use such services for free or reduced prices at the point of consumption. In other words, a comparison of both perspectives (supply and demand) shows that, although the market production of health care activities in the whole of the EU and EA registers the greatest weight, financing these health care services is mostly public.

#### Final consumption expenditure of general government

Table 4 provides information extracted from the use table at purchasers’ prices for different countries (these tables are not available for the whole of the EU or for the EA), on their GG’s FCE on health care services and its relationship with GDP and with the total FCE of the GG.

**Table 4:** Final consumption expenditure of general government (GG FCE) on health care services (% and PPS EU28) (use table, purchaser prices, domestic and imports) 2010

	GG FCE on health care services (% of GDP)*	GG FCE on health care services (% of Total GG FCE)**	GG FCE on health care services per capita (in PPS_EU28)***		GG FCE on health care services (% of GDP)*	GG FCE on health care services (% of Total GG FCE)**	GG FCE on health care services per capita (in PPS_EU28)***
EU-28	:	:	:	Latvia	2.4	12.9	485.4
EA-19	:	:	:	Lithuania	3.7	18.7	711.5
Belgium	5.1	21.8	1,409.6	Luxembourg	2.5	14.4	1,127.5
Bulgaria	2.1	13.0	420.5	Hungary	3.1	14.3	784.7
Czech Rep	4.5	22.2	1,317.5	Malta	4.5	23.1	856.1
Denmark	5.7	20.7	1,751.6	Netherlands	4.8	17.9	1,357.0
Germany	4.8	25.3	1,520.2	Austria	4.8	23.6	1,378.9
Estonia	3.7	18.2	775.4	Poland	3.1	16.0	690.9
Ireland	6.2	33.7	1,477.1	Portugal	4.6	22.1	813.5
Greece	3.6	16.1	729.3	Romania	3.8	24.2	841.5
Spain	4.7	22.9	985.9	Slovenia	4.4	21.7	974.5
France	5.3	22.4	1,441.4	Slovakia	2.9	15.3	881.5
Croatia	:	:	:	Finland	5.2	21.6	1,482.0
Italy	5.7	27.7	1,381.5	Sweden	5.6	22.3	1,504.3
Cyprus	2.4	13.0	572.4	UK	6.6	30.8	1,895.5

^a^Coefficient of variation: (*) 0.29; (**) 0.26; (***) 0.37

^b^(:) not available

*Source:* Prepared by authors with Eurostat data, "Use table at purchasers' prices (domestic and imports)"/"Purchasing power parities (PPPs)"/"Euro/ECU exchange rates—annual data"/"Population on 1 January by age and sex"

The weight of GG expenditure with respect to GDP ranges from maximum values greater than 6% in the United Kingdom and Ireland to minimum ones of less than 3% recorded in Slovakia, Luxembourg, Latvia, Cyprus and Bulgaria (with a coefficient of variation of 0.29). Regarding the weight that health care services’ FCE represents in the total FCE of GG, although it has a slightly lower coefficient of variation (0.26), it also records a fairly wide range of values, from 33.7% in Ireland to 12.9% in Latvia.

The third column of Table 4 presents the FCE of the GG in per capita health care services in PPS for each EU country. The table reveals the notable differences in the EU in terms of public financing per capita of health care services (coefficient of variation of 0.37, significantly higher than the two mentioned above). The difference between the two extreme countries shows that the real expenditure (PPS) per capita of the GG in health care services in the United Kingdom is 4.5 times greater than that corresponding to Bulgaria.

#### Households’ final consumption expenditure

For the data to be comparable with those provided in the rest of this section, the information presented in Table 5 has been extracted from the corresponding use tables at purchasers’ prices. Household FCE in health care services with respect to total Household FCE ranges in weight in the different countries of the European Union ranging from a minimum of 0.6% in Luxembourg to the maximum 3.6% in Greece. Regarding Household FCE in per capita health care services in PPS for each of the countries, the information reveals a greater variability than that observed previously for the FCE of the GG in the same type of services (coefficient of variation of 0.41), increasing the difference between the two extreme countries: the real expenditure (PPS) per capita of Households in health care services in Cyprus is 6.6 times greater than that corresponding to Lithuania. The information recorded by the different tables of the input–output framework for Cyprus seems to show a somewhat peculiar situation. Although the participation of its market health care activities in the total supply of such activities is below that of the EU as a whole (44.1% vs. 54.4%), the weight of Households’ FCE in this type of services is the highest (47.2%) of all EU countries, and much higher than the indicator registered by all such countries (EU 28), which is 17.3%. This could indicate that, in this case, households are contributing significantly to finance the supply of these market health care services, or, in other words, that market STIK financed by the GG of that country are very scarce or practically non-existent. A very similar situation occurs in Greece: supply of 51.5% by market health care activities and FCE weight of Households in aggregate demand of 38.1%, the second highest after Cyprus.^44^

**Table 5:** Final consumption expenditure of households (HH FCE) on health care services (% and PPS_EU28) (use table, purchasers' prices, domestic and imports). 2010

	HH FCE on health care services (% of Total HH FCE)*	HH FCE on health care services per capita (in PPS_EU28)**		HH FCE on health care services (% of Total HH FCE)*	HH FCE on health care services per capita (in PPS_EU28)**
EU-28	:	:	Latvia	1.96	255.50
EA-19	:	:	Lithuania	0.64	79.39
Belgium	3.00	410.92	Luxembourg	0.56	93.55
Bulgaria	1.62	219.00	Hungary	2.09	282.93
Czech Rep	1.11	164.02	Malta	1.79	223.44
Denmark	1.60	228.03	Netherlands	2.04	253.86
Germany	3.10	518.13	Austria	2.13	328.49
Estonia	1.14	129.52	Poland	1.59	217.49
Ireland	3.11	336.06	Portugal	3.15	372.65
Greece	3.58	514.75	Romania	1.81	245.29
Spain	2.30	282.43	Slovenia	1.81	234.56
France	2.39	352.58	Slovakia	1.47	252.25
Croatia	:	:	Finland	2.65	383.15
Italy	1.82	272.38	Sweden	1.85	221.50
Cyprus	3.21	520.43	UK	1.38	245.30

^a^Coefficient of variation: (*) 0.39; (**) 0.41

^b^(:) not available

*Source:* Prepared by authors with Eurostat data, "Use table at purchasers' prices (domestic and imports)"/"Purchasing power parities (PPPs)"/"Euro/ECU exchange rates—annual data"/"Population on 1 January by age and sex"

#### Total final consumption expenditure (GG and Households)

**Fig. 7:**
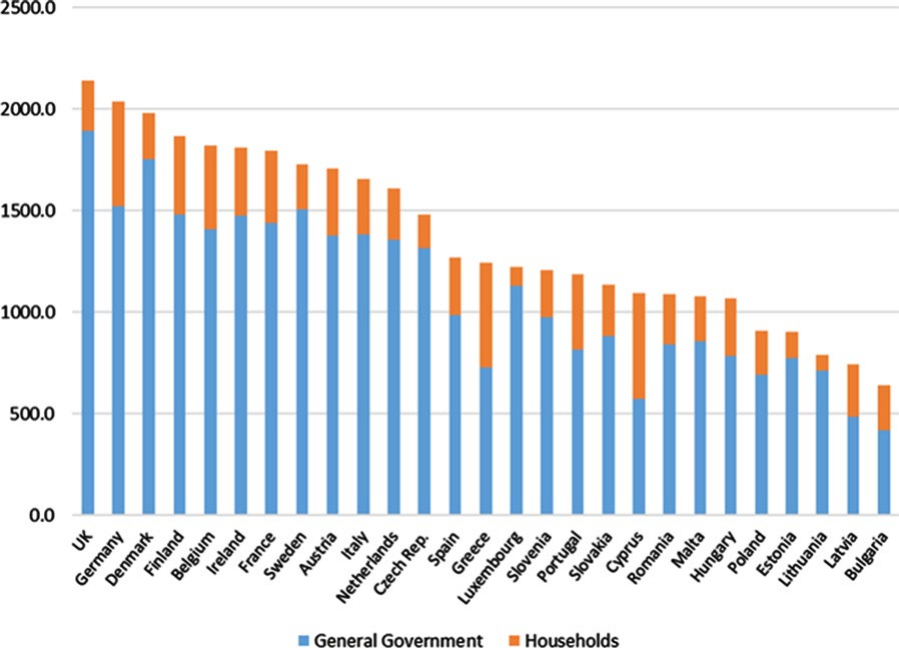
Total Final Consumption Expenditure (GG and Households) per capita on health care services (in PPS_EU28) (Households actual final consumption approach). 2010. *Source:* Prepared by authors with Eurostat data "Use table at purchasers' prices (domestic and imports)"/"Purchasing power parities (PPPs)"/"Euro/ECU exchange rates—annual data"/"Population on 1 January by age and sex"

Finally, in Fig. 7, the total expenditure (PPS) per capita (Households and General Government) on health care services is presented. This is an approximation of the actual final consumption in health care services by household. Although, as already mentioned, in the per capita expenditure of both institutional sectors, important differences were observed between different countries, these differences are partially cushioned when evaluating the total FCE per capita, reducing the coefficient of variation to 0.31. Moreover, the difference between the two extreme countries shows that the country with the highest real expenditure (PPS) per capita total in health care services (United Kingdom) is 3.3 times greater than the lowest indicator recorded by Bulgaria.

**Fig. 8:**
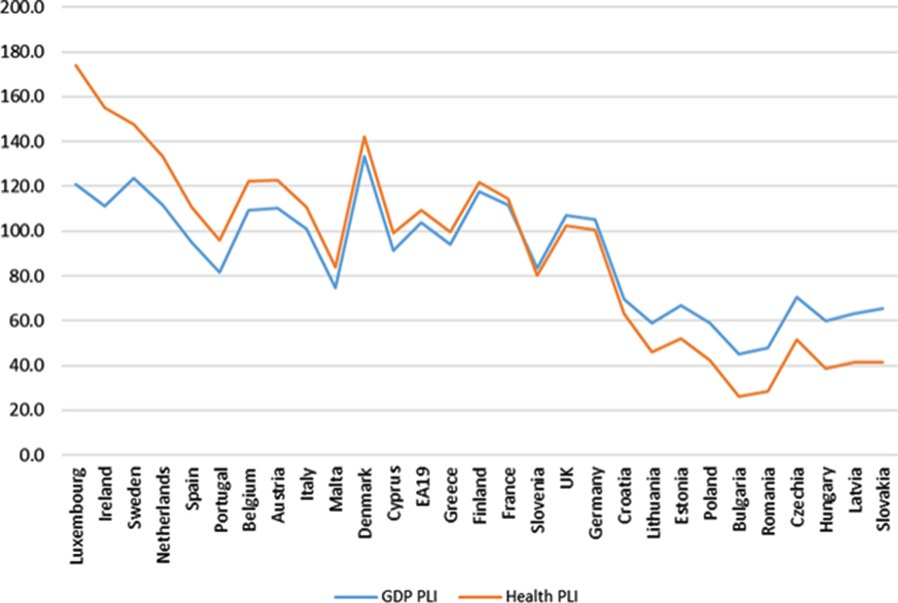
Comparison of Price level indices (PLI) for GDP and Health (Index EU28 = 100). 2010. *Source:* Prepared by authors with Eurostat data, "Purchasing power parities (PPPs)"/"Euro/ECU exchange rates—annual data"

It is common that in many published works comparing health care expenditure between countries, purchasing power parities (PPPs) referring to the total economy (GDP) are used as a conversion factor and not those specific to “health,” as has been done in this study. Both alternatives can generate results that, at times, are quite different because of the differences between the price levels of health services and between general price levels (GDP) in each country, as shown in Fig. 8, in which such differences are presented by calculating the price level indices (EU = 100).^45^

In the majority of countries (15), the price level of health care in relation to the average level for such services in the EU is higher than that recorded in the general price level regarding the average EU level. In addition, it is found that, at low levels of the indices, the general price level is above the price level for health care, while at high levels, the opposite is true. On the other hand, the relative variation in general price levels between countries (GDP) is markedly smaller than that recorded for health care prices (coefficients of variation of 0.284 and 0.461, respectively).

**Fig. 9:**
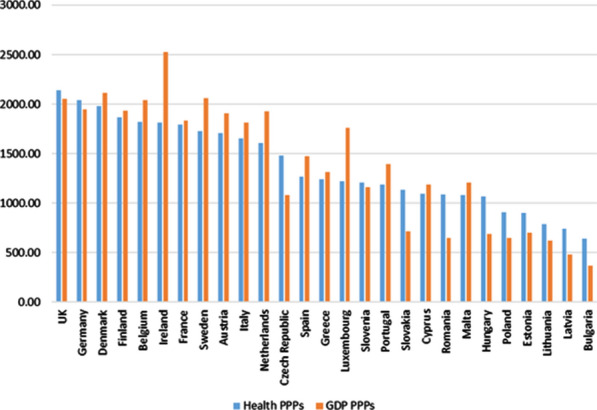
Comparison of Total Final consumption expenditure (GG and Households) per capita on health care services (in PPS_EU28), using PPPs for GDP and for Health (Households actual final consumption approach). *Source:* Prepared by authors with Eurostat data, "Use table at purchasers' prices (domestic and imports)"/"Purchasing power parities (PPPs)"/"Euro/ECU exchange rates—annual data"/"Population on 1 January by age and sex"

The results for total final consumption expenditure (General Government and Households) obtained by applying both alternatives are presented in Fig. 9.

Thus, if we had considered PPPs for GDP instead of health-specific PPPs, in countries where the price level (compared to the EU 28 average) for health care services exceeds the general price level, as, for example, occurs in Ireland, Sweden, the Netherlands or Luxembourg, it would appear that they record a total FCE (Households + GG) per capita higher than what is actually produced. On the other hand, countries in which the situation regarding price levels is the opposite, such as, Czech Republic, Slovakia, Romania or Hungary, it would appear they had a lower total FCE (Households and GG) per capita than occurs in reality. As a result, the ranking of countries according to FCE changes considerably depending on the PPP used (20 out of 27 countries change the position in the ranking when the type of PPP is changed). The “homogenizing” effect, caused by the use of health-specific PPPs, can be seen by checking that the coefficient of variation of the total FCE per capita in health care services, when PPPs are used for GDP, increases up to 0.45 value (vs. 0.31). In addition, the difference between countries with higher and lower total FCE per capita (Ireland and Bulgaria) also increases, multiplying by 6.8 the lowest indicator (3.3 with PPPs for health).

### An approximation to apparent labour productivity in health care activities

**Fig. 10:**
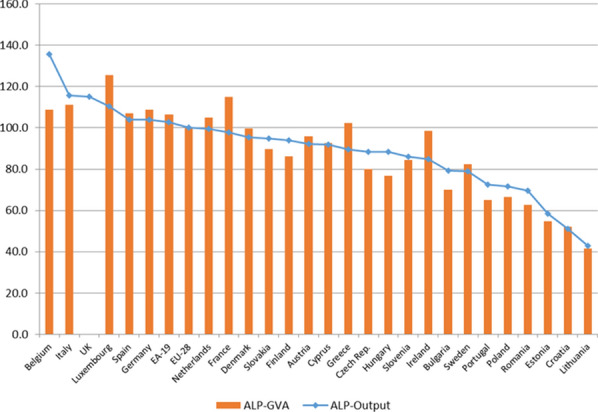
Health care activities. Apparent labour productivity (ALP) (in PPS_EU28 per hours worked) (Index EU28 = 100). 2010. *Source:* Prepared by authors with Eurostat data, "Supply table at basic prices"/"Use table at basic prices (domestic)”/“National Accounts employment data by industry”/"Purchasing power parities (PPPs)"/"Euro/ECU exchange rates—annual data"

In Fig. 10, the apparent labour productivity is presented for all the countries for which the necessary data are available, taking as a numerator the two aggregates already mentioned (output and GVA), valued (health PPPs) in PPS (EU28), in the form of indices (EU28 = 100). The existence of differences in the apparent labour productivity in the development of health care activities can be seen between different countries. Productivity in countries like Luxembourg, Italy or Belgium almost doubles that of countries such as Lithuania, Croatia or Estonia. In addition, the coefficients of variation are 0.24, when the GVA is used, and 0.22 when the output value is used.^46^

These differences in apparent productivity may be due, at least in part, to the different weight that market or non-market output has in the total output value in different countries. This is because the calculation of the value of both types of production (as well as that of generated GVA), according to the method used in national accounting, can cause some distortions.

As stated above, the value of the production of health care activities can be obtained by adding the intermediate consumption used and the GVA generated, mainly consisting of employees’ compensation and the gross operating surplus (GOS). The difference between the value of market production and that of non-market lies in the GOS component, made up of, in turn, by net operating surplus (NOS) and consumption of fixed capital (CFC). In the case of non-market production, the GOS coincides with the CFC, the NOS being zero. Obviously, this distortion also translates to the calculation of the GVA.

**Fig. 11:**
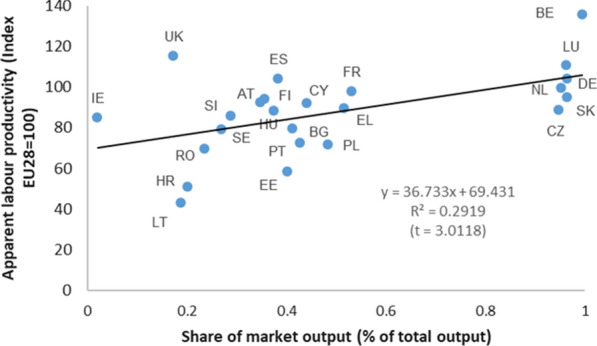
Health care activities. Relation between apparent labour productivity (in PPS_EU28; Index EU28 = 100) and the share of market output (% of total output). 2010. *Source:* Prepared by authors with Eurostat data, "Supply table at basic prices"/"Use table at basic prices (domestic)”/“National Accounts employment data by industry”/"Purchasing power parities (PPPs)"/"Euro/ECU exchange rates—annual data"

Figure 11 shows the relationship between the productivity indicator and the percentage that represents the market production in health activities.

There seems to be a positive and significant relationship between both variables. In addition, it is observed how the group of countries in which the weight of the market output is close to 100% (Belgium, Luxembourg, Germany, Holland, Slovakia and the Czech Republic), all have productivity rates above the European average. Some exceptions to this rule are Ireland, the United Kingdom or Spain, where, despite non-market output registering a greater weight, they have productivity rates that are much higher than expected according to the established relationship. This relationship is evident both when the value of the output is used to calculate productivity and when the GVA is used. When the indicators refer to total economic activities of different countries (with PPPs for GDP), the relationship is less intense, and only exists when the output value is used.

**Fig. 12:**
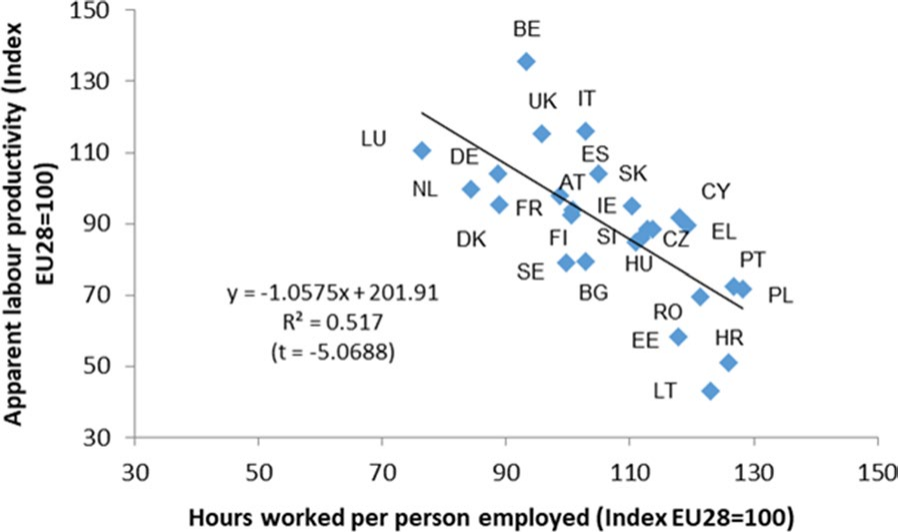
Health care activities. Relation between apparent labour productivity (in PPS_EU28; Index EU28 = 100) and hours worked per person employed (Index EU28 = 100). 2010. *Source:* Prepared by authors with Eurostat data "Supply table at basic prices"/"Use table at basic prices (domestic)”/“National Accounts employment data by industry”/"Purchasing power parities (PPPs)"/"Euro/ECU exchange rates—annual data"

On the other hand, Fig. 12 shows the relationship between the apparent productivity indicator and the number of hours worked per person employed in health care activities. In general, the countries in which the number of hours worked per employee in these activities is the lowest, are those with the highest levels of apparent labour productivity (measured by output and in PPS, in the form of indices, EU = 100). This relationship is maintained when productivity is calculated using the GVA of health care activities. It has also been concluded that the relationship is maintained when the above indicators refer to the set of industries of different economies (with PPPs for GDP). It seems that countries in which the number of hours worked per person employed in health care activities (or in all industries) is lower show higher apparent labour productivity.

In order to provide some additional evidence on the relationships suggested by Figs. 11 and 12, several hypotheses have been tested (the results of the regressions are shown in Table 6).

**Table 6:** Regression results

	Number of countries	Excluded countries (not available)	Dependent variable (y)	Independent variable (x)	β		t-Test
***Health care activities***	***26***	Latvia, Malta	***ALP-Output (index)***	***Hours worked per person employed (index)***	− 1.0575	***	− 5.0688
*All industries*	*27*	Latvia	*ALP-Output (index)*	*Hours worked per person employed (index)*	− *2.2483*	***	− *3.4947*
***Health care activities***	***25***	Latvia, Malta, UK	***ALP-GVA (index)***	***Hours worked per person employed (index)***	− 1.1888	***	− 5.6732
*All industries*	*26*	Latvia, UK	*ALP-GVA (index)*	*Hours worked per person employed (index)*	− *2.1473*	***	− *5.1287*
***Health care activities***	***24***	Denmark, Italy, Latvia, UK	***GOS (% GVAfc)***	***Market output (%)***	20.2157	**	2.8061
***Health care activities***	***23***	Denmark, Italy, Latvia, Malta, UK	***GOS (PPS_EU28 per hours worked)***	***Market output (%)***	8987.8030	***	3.7132
***Health care activities***	***24***	Denmark, Italy, Latvia, Malta	***Hours worked per person employed (index)***	***Market output (%)***	− 23.4032	**	− 2.7455
***Health care activities***	***23***	Denmark, Italy, Latvia, Malta, UK	***ALP-GVA (index)***	***Market output (%)***	39.1176	***	2.8920
***Health care activities***	***24***	Denmark, Italy, Latvia, Malta	***ALP-Output (index)***	***Market output (%)***	36.7325	***	3.0118
*All industries*	*26*	Denmark, Latvia	*ALP-Output (index)*	*Market output (%)*	*4.3266*	*	*1.8268*

*Source:* Prepared by authors with Eurostat data, "Supply table at basic prices"/"Use table at basic prices (domestic)”/“National Accounts employment data by industry”/"Purchasing power parities (PPPs)"/"Euro/ECU exchange rates—annual data"

***, **, * Significance level of 1%, 5% y 10%, respectively

^a^ALP-Output (VAB) (index): Output (GVA) in PPS_EU28 per hours worked (index EU28 = 100)

^b^Market output (%): Share of market output (% of total output)

^c^Health PPPs for health care activities and GDP PPPs for all industries

^d^GOS (% GVAfc): Gross operating surplus (% GVA at factor cost)

First, countries in which the weight (% of total output) of market production in health care activities is greater, also recorded a greater weight of GOS in the GVA at factor cost (that is, without considering possible taxes or subsidies on production). By contrast, countries in which the weight of non-market output in health care activities is greater, also have a greater weight of employees’ compensation in GVA. If instead of the weight of the GOS in the GVA, the GOS (in PPS) per hour worked in the health care activities is taken, the positive relationship with the weight of the total output of the market output is strengthened, while the relationship between the compensation of employees (in PPS) per hour worked and the weight of non-market output in health care activities is weakened. If the above calculations are made for the same indicators but refer to the total economic activities of the different countries, the relationship is apparently weaker, and the absence of a relationship cannot be ruled out.

Second, countries in which the weight (% of the total output) of market output in health care activities is greater also record a smaller number of hours worked per person employed in these activities. By contrast, the countries in which the weight of non-market output in health care activities is greater record a larger number of hours worked per person employed. The absence of such effects cannot be ruled out when the indicators refer to the total economic activities of the different countries.

To sum up the results obtained, those countries with the greatest weight of market output in health care activities have a lower number of hours worked and a higher productivity per hour worked. In addition, those countries with the greatest weight in market output also have a greater weight of GOS in the GVAfc (factor cost) and a greater amount of GOS (in PPS) per hour worked. Since the GOS has different components that depend on whether health care activities are carried out as a market or non-market activities, the lower weight (and lesser amount) of GOS in countries with greater non-market output weight could also justify, at least partially, a lower production (and GVA) value and a lower apparent labour productivity in health care activities. In other words, it can be concluded that, at least partially, the relationship observed in Figs. 11 and 12 could be a consequence of the different components that integrate the value of production in market or non-market health care activities, regardless of whether they can be developed with greater or lesser apparent labour productivity. This reasoning cannot be extended to all activity industries.

## Discussion

The comparison of perspectives of supply (supply table) and demand (use table) under the input–output framework makes it possible to state that in the EU as a whole, health care activities output is mostly produced by the market and mostly financed by the public sector. However, comparisons between countries show that there is significant variability, and, in fact, in most countries, non-market output has the greatest weight. There is also significant variation between countries in total final consumption expenditure (GG and Households) per capita in health care services, with the component financed by households being the one that most causes this variability (there is greater homogeneity in the component financed by General Government).

Regarding the interrelation of the health care sector with the productive activities of the economy in the European Union, when measured at basic prices, health care activities have weak and below average direct backward linkages for all industries of the economy and below the average of the service industries. These linkages are formed, for the most part, by the following intermediate inputs: health care services themselves (intra-consumptions, such as external market diagnostic tests or the hiring of self-employed professionals), wholesale and retail trade services, in-hospital pharmaceutical products, and administrative and support services. When measured at purchasers’ prices, it is striking that, in most countries, the weight of pharmaceutical products over total intermediate consumption increases significantly (with respect to basic prices), around four or five percentage points, reflecting the significant weight of distribution margins (trade and transport) and taxes on pharmaceutical products. As regards direct forward linkages, the use of health care services is primarily oriented towards final demand, which means that the use of such services by the producing industries is of a residual nature, showing even weaker forward linkages that are smaller than the average for all types of products and the average of services. The intermediate products with greatest weight in these forward chains represent intra-consumptions.

In the comparison between countries, some variability can be seen in both backward and forward linkages. Especially striking is the case of Belgium, which has the greatest weight of intermediate inputs and intermediate outputs. In addition, this greater interrelation is concentrated in the health care sector itself, since intra-consumption represents the greatest weight among intermediate inputs and outputs, which reflects (in other countries, such as, Poland, Portugal or Spain) a greater degree of interrelation between producers of health care activities and services.

Given that direct linkages in both directions are below average in practically all the countries analysed, it could be said that the health care sector (health care activities and services) are relatively independent sectors in the productive structure of these economies.

The simple output multipliers of health care activities record values significantly lower than the average multiplier for all industries, which is consistent with the weak backward linkages already detected, adding evidence of the weakness of the indirect effects (not only direct) on the whole economy. Again, as was the case with backward linkages, Belgium is at the top of the output multipliers, while France is at the bottom (also with backward linkages).

The low use of intermediate inputs by the industry of health care activities contrasts with the enormous weight of the GVA generated per unit of industry output, higher than the average for all industries and also higher than the service industries average (with the only exceptions of Belgium and Hungary). Therefore, in all the countries analysed, health care activities could be considered as key because of their ability to directly generate (in the industry itself) value added in the economy. In addition, the final demand for health care services has the capacity to generate (directly or indirectly) in the whole economy more value added than the average for all types of products.

With regard to employment in health care activities, there are two aspects that have focused the attention of this study. First, the ability to generate employment (measured as the requirement of hours worked per unit of output) and, second, the apparent labour productivity. With respect to the former, the number of hours required directly per unit of output in health care activities is higher than the average for all industries of the economy (highlighting the greater capacity for generating employment in Denmark, France, Hungary or the Netherlands). When we make the same calculation, but considering the total number of hours worked (not only direct but also indirect), the greater relative capacity to generate employment in the whole economy is maintained (although with less intensity) when the final demand for health care services increases, in particular in Hungary, Denmark and Croatia.

As regards the apparent labour productivity in health care activities, our results indicate that there are considerable differences between EU countries. The apparent productivity in countries such as Luxembourg, Italy or Belgium almost doubles that of countries such as Lithuania, Croatia or Estonia, and is significantly superior to that of others like Sweden, Portugal or Poland. Some of these differences may have two origins. First, they may be due to a simple accounting effect. Countries with greater relative weight of market producers (which are precisely those with highest labour productivity rates) can register a higher value of production simply because their net operating surplus is not zero, as occurs in non-market producers. In other words, for the same health care activity carried out under the same conditions (same amount and compensation for employees, same consumption of fixed capital and identical use of intermediate consumption, as well as identical provision for the rest of production factors), the value of its production (and GVA) will be different. It will be lower if it is made by a non-market producer than if it is carried out by a market producer, which would result in greater apparent labour productivity in the case of the activity developed by a market producer. Schreyer and Mas (page 3) [27] present a similar reasoning. Second, these differences in apparent productivity could also be explained by the hypothesis that labour productivity is negatively related to the number of hours worked per person employed. Our results suggest that the countries with the lowest labour productivity are those with the highest number of hours worked per person employed in health care activities. In addition, we observe that the countries with the greatest weight of market production in health care activities are those with the least number of hours worked per person employed, strengthening the explanation of at least part of the variability in the apparent labour productivity that we find in the EU countries. We are aware that in the regression analysis undertaken, the sample size is small, so the distributional assumptions used to carry out significance tests can be questionable. However, we must also say that not much can be done regarding sample size, as there were just 28 countries in the EU in 2010. On the other hand, from a descriptive point of view, we consider that the analysis remains valid and sufficiently clear to draw the main conclusions reached. In any case, it would be necessary to analyse more deeply the reasons or causes that could justify the existence of this inverse relationship between greater apparent labour productivity and fewer hours worked per person in health care activities of market or non-market output, an issue that could be addressed in future research.

Finally, some research limitations should be taken into account. First, there may be certain considerations that could limit the comparability of measurements of hospital services, with other services, such as education, whose provision is mainly by the GG (non-market producers). Schreyer and Mas [27] among others, mention their doubts about whether the definition of health care services is the same between countries, or if the methodologies applied by statistical offices to perform the decomposition between prices and volumes over time are similar. In this regard, they indicate that international comparisons of health care expenditures should be based on measures that reflect the cost of providing such services, regardless of whether that cost was borne by households, by market or non-market producers, positively assessing the new approach developed by Eurostat-OECD to estimate specific PPPs for health. Second, we have not been able to undertake the analysis of the productive structure and intersectoral relations of the health care sector for all the member countries of the European Union. The non-existence of some input–output tables for certain countries, as well as the errors detected in some of the available tables, have therefore limited the comparison analysis within the EU. These limitations are specified in Annex 2.

## Conclusions

In the EU as a whole, the output of health care activities is mostly financed by the public sector and mostly produced by the market (although in most countries, non-market output has the greatest weight). In addition, the health care sector in the EU and in the countries that integrate it are relatively independent in the productive structure of their economies, as shown by the weakness of both backward and forward linkages of the health care sector with the rest of the economy. This contrasts with the remarkable ability of the health care sector to create value added and employment. Regarding apparent labour productivity, there are significant differences among EU countries, showing that productivity is positively related to the weight of market production in health care activities and negatively related to the number of hours worked per person employed. To compare health care expenditure between countries, data in national currencies have been adjusted using a conversion factor that is the specific purchasing power parities for “health”, and not one referring to the total economy (GDP), as is common in many previous studies. These specific purchasing power parity for health is also used to compare apparent labour productivity among countries. The results when using distinct purchasing power parities are very different, and this should be taken into account when interpreting our results.

## Supplementary Information

**Additional file 1.** Methodological summary of input-output analysis (.doc). Additional file 1 contains a methodological summary of the input-output framework and the analysis applied in this study.

**Additional file 2.** Sources of information (.doc). Additional file 2 explains in detail the sources of information used in this study

**Additional file 3.** Correspondence table of industries/products with the Eurostat input-output framework and the NACE rev. 2/CPA 2008 (.doc). Additional file 3 contains a table with industries/products and their respective Eurostat input-output framework and the NACE rev. 2/CPA 2008.

**Additional file 4.** Complementary tables and figures (.doc). Additional file 4 contains eleven tables with complementary information on health care services, health care activities, direct and indirect requirements for GVA and for hours worked. And it also contains one figure with a comparison of apparent labour productivity on health care activities using PPPs for GDP and for health.

## Data Availability

Data are available from the EUROSTAT website (http://ec.europa.eu/eurostat/data/database).

## Abbreviations

ALP: Apparent labour productivity
Bp: Basic prices
CFC: Consumption of fixed capital
COFOG: Classification of the functions of Government
CPA: Statistical Classification of Products by Activities
EA: Euro area (19 countries)
ESA: European System of Accounts
EU: European Union (28 countries)
Fc: Factor cost
FCE: Final consumption expenditure
GCF: Gross capital formation
GDP: Gross domestic product
GG: General Government
GOS: Gross operating surplus
GVA: Gross value added
HH: Households
NACE: Statistical Nomenclature of Economic Activities
NOS: Net operating surplus
NPISH: Non-profit institutions serving households
PLI: Price level indices
PPP: Purchasing power parities
PPS: Purchasing power standard
SHA: System of Health Accounts
SIOT: Symmetric input–output table
SNA: System of National Accounts
STIK: Social transfers in kind

## Footnotes

1 Details of them can be found in Chapter 3 (pp. 33–49) and in Annexes A (pp. 379–414) and B (pp. 415–442) of OECD, Eurostat, WHO [4].

2 Additional file 1 includes a methodological summary of the input–output framework and the analysis that has been applied in this paper.

3 On the process of construction and aggregation of the different tables for the European Union, see Rueda-Cantuche, J. et al. [6], Eurostat & Joint Research Center [7], Duguleana, L. and Duguleana, C. [8].

4 The relation of economic activities (industries) considered is based on the Statistical Nomenclature of Economic Activities (NACE Rev.2) [9]. The industry analysed in this work corresponds to division 86 of the aforementioned nomenclature, whose specific designation is “human health activities”.

5 The principal activity is considered the one whose value added exceeds that of any other activity carried out by the same producer.

6 For example, social counseling or guidance services provided in hospitals by certain official bodies or private organizations.

7 A secondary activity is an activity carried out by a producer in addition to the main activity. The result of a secondary activity is a secondary product.

8 This is the case, for example, of consultation and treatment activities carried out by doctors in nursing homes for the elderly.

9 The list of products considered is based on the *Statistical Classification of Products by Activities* (CPA 2008) [10]. Health care services correspond to division 86 of the classification, whose specific designation is “human health services”.

10 In the ESA [11], uses of products are valued at purchasers’ prices, which include transport costs, trade margins and taxes less subsidies on products, while output is recorded at basic prices, which exclude these elements. In other words, production at basic prices would add to the production costs (intermediate consumption and the compensation for labor and capital) the so-called other taxes on production and deduct other subsidies on production, which usually represent a low relevance.

11 Additional file 2 details the data sources that have been used as well as the limitations of such data (especially the fact that not all tables present information for the same countries or for the same number of countries). Moreover, Additional file 3 contains the correspondence table of industries/products with the Eurostat input-output framework and the NACE rev. 2/CPA 2008.

12 The supply of secondary products by an industry is calculated with the information contained in the columns of the supply table, by means of the so-called specialization coefficients (quotient between the supply of each type of product and the total output): this would represent the percentage of the supply of secondary products over the total production of the industry, the rest corresponding to the supply related to the principal activity (product).

13 For countries where there are use and SIOT tables (at basic prices), certain differences have been detected in the Czech Republic, Greece and Slovakia.

14 See Additional file 1 regarding methodology on input–output.

15 That is to say, the one arising directly from the acquisitions of intermediate inputs by this industry in order to be able to carry out its production, as well as the production that is generated indirectly as a result of the acquisitions of intermediate inputs that the suppliers of health care activities must perform to be able to supply the inputs needed.

16 Hours worked correspond to the number of hours actually worked and includes the sum of all periods dedicated to direct and auxiliary activities for the production of goods and services. Travel time between home and workplace is excluded, as well as main meal breaks.

17 This information does not coincide exactly with that obtained from the SIOT, since the number of hours worked here is used to refer to industries, while the production recorded in the SIOT refers to products.

18 Capital expenditures are not included (gross capital formation and capital transfers, primarily).

19 The rest would correspond to the collective consumption expenditure. For the products supplied by the General Government, the limit between individual and collective services is established in accordance with the Classification of the functions of Government (COFOG) [20], although this information is not comparable to that offered by the different tables of the input -output framework in their rows (in this case, in the row of health care services), since it integrates expenditure into a series of activities or products that fall outside the scope of the services defined by health care according to the NACE [9] and the CPA [10]. The collective FCE of the General Government corresponding to the Health function (No. 7 of COFOG) is mainly composed of the expenses in general administration, legislation, dissemination of general information and statistics, as well as the expenses in research and development.

20 To evaluate the financing of medicines supplied directly to households on an outpatient basis, information collected in the row of the use table or the SIOT corresponding to the basic pharmaceutical products and their preparations should be incorporated.

21 The FCE of Households presented here (and the one that the use table or SIOT provide in the different rows for each product) corresponds to that made in the economic territory, and, therefore, includes that made by resident and non-resident households.

22 In the EU and EA, the number of hours worked per person employed in health care activities (1,557.2 and 1,514.1, respectively) was lower in 2010 compared to the set of all industries (- 5.6% and -5.0%, respectively).

23 Although the total number of industries considered in this work is 37, of which 19 produce services, for the calculation of the average (sum of the backward linkages from the industries divided by the number of industries in each case), industry No. 37 (Activities of households as employers: undifferentiated goods and services producing activities of households for own use) has been excluded, since it does not use intermediate consumption (its direct backward linkage is null) and thus distorts the average for the industries. Therefore, the total numbers of industries considered are 36 and 18, respectively. However, the tables provided in Additional file 4 also include averages calculated without excluding industry No. 37.

24 According to the ESA 2010 [11], goods and services produced and consumed during the same accounting year within the same unit of local economic activity are excluded from this calculation, that is, using the terminology of the 2008 SNA [1], within the same establishment. On the other hand, since the production of non-market health care services is individual and its only possible use, according to the ESA [2, 3], is final consumption expenditure of general government, these “intra-consumptions” could only be integrated by market production of health care services used in the health care activities (market or non-market) provided that they do not constitute social transfers in kind (concept that will be studied later), whose use would also be for final consumption expenditure. As examples, these "intra-consumptions" can consist of the purchases of external (market) health entities of diagnostic tests (diagnostic imaging, blood tests, etc.) or by hiring their own professional health workers.

25 Table S6 of Additional file 4 provides a similar table drawn up from the SIOT (product by product) for the countries in which this information is available, verifying that the differences with respect to the data obtained from the use table are very insignificant.

26 In the input–output framework for the EU or the EA as a whole, foreign trade in goods and services exclusively includes that carried out with countries that are not members of the EU or EA, respectively, since that carried out among the different members has the character of “domestic”. For each EU or EA member country, foreign trade includes that made with any other country, whether it is an EU or EA member, respectively. See Bouwmeester, M. C. et al. [4].

27 The data at basic prices on weights (domestic and imports) for each type of product in each of the countries analyzed are presented in Table S8 of Additional file 4.

28 In the EU, imports of these products (non-EU origin) accounted for 24.8% of total supply at basic prices in 2010 (32.8% in the EA). These weights show great variability between countries. For example, the following: Denmark, 29.4%; Germany, 49.7%; Ireland, 14.6%; Greece, 74.7%; Spain, 62.2%; Lithuania, 89.3%; Netherlands, 81.8%; Poland, 63.9%; or, United Kingdom, 46.9%.

29 The same applies to the industries of education and social work activities, although, in the former, these requirements are lower than those of health care activities, while in the latter the situation varies between countries.

30 The information recorded in the supply table (basic prices) of the 28 EU member countries shows that in 25 countries the imports of health care services represent between 0 and 1% of the total supply (internal and imported) of such services. Only three countries exceed that weight: Bulgaria (2%), Luxembourg (3.7%) and United Kingdom (1.4%).

31 Goods and services for individual consumption (“individual goods and services”) are goods and services acquired by a household and used to satisfy the needs and wants of members of that household. Collective services are services for collective consumption that are provided simultaneously to all members of the community or all members of a particular section of the community.

32 Something similar occurs with other services (products) of an individual nature such as education or social work services, although in the latter case, for most of the countries analyzed, the weight of intermediate outputs in the total of possible uses is even lower.

33 Averages have been calculated as the sum of the forward linkages of the products considered (37 in the case of total products or 19 in the case of services) divided by the number of products in each case.

34 The calculations have been made based on data on intermediate consumption at basic prices and of domestic origin, because, as already indicated above, it is about assessing the impact that is generated on the production of the corresponding territory or country, thus eliminating imports of intermediate inputs, i.e., possible multiplier effects that could be from abroad (other territories or countries).

35 The details of the indirect effects caused by health care services on the production of each product considered are presented in Table S10 of Additional file 4.

36 As already indicated, industry No. 37 does not use intermediate consumption, so the value of its production coincides with the GVA, and the corresponding ratio has a value of 1. Therefore, to avoid the possible distortion that this high value could generate, in the calculation of the averages (total industries or service industries) the information of said industry has been excluded. However, in Table S11 of Additional file 4, the averages are also provided without excluding industry No. 37. This same table in Additional file 4 also presents all the basic information used in Fig. 3, as well as the same indicators obtained from the information contained in the SIOT for those countries in which this information is available. This verifies that the differences with those calculated from supply or use tables are minimal.

37 The same applies to other industries that produce services of an individual nature, such as education or social work activities. In the first case, the GVA generated directly in all the countries analysed is higher than that registered by health care activities, while in the second, the situations differ between countries. For the EU altogether, these coefficients have the values ​​of 0.80 and 0.66, respectively (0.82 and 0.72 in the EA).

38 Table S12 of Additional file 4 shows the basic information used to prepare the figure on the total GVA generated, as well as the same indicators obtained from the information contained in the SIOT for those countries in which said document is available, thus being able to verify that the differences with those calculated from the supply or use tables are minimal.

39 Given that in industry No. 37, the value of production coincides with the GVA (does not use intermediate consumption), the number of hours worked in the production of this industry is oversized. Therefore, to avoid the possible distortion that this high value could generate, in the calculation of the averages (total industries or service industries) the information of this industry has been excluded.

40 As can be seen later in this section (Fig. 8. Comparison of Price level indices (PLI) for GDP and Health), the three countries mentioned are part of the group of EU countries that have lower price levels for health.

41 The same situation occurs in all countries with the industries of education and social work activities, in both cases with ratios higher than those registered for health care activities.

42 According to the ESA 2010 [12], market output consists of output that is disposed of on the market or intended to be disposed of on the market (at economically significant prices); Output produced for own final use consists of goods or services that are retained either for own final consumption or for capital formation by the same institutional unit; Non-market output is output that is provided to other units for free, or at prices that are not economically significant. According to the ESA, economically significant prices are those that, applied to sales, cover, continuously or over several years, at least 50% of the production costs of the producing unit (sum of intermediate consumption, compensation of employees, consumption of fixed capital and other taxes less other subsidies on production). Non-market production can be developed exclusively by non-market producers (General Government -GG- or non-profit institutions serving households –NPISH-). Market production and for own final use can be developed (as principal or secondary activity, depending on the case) by any type of producer. Non-market production is calculated from the sum of production costs.

43 In the ESA 2010 [13], an important part of the output produced for final use of the health care activities industry corresponds to research and development costs, including the production of R&D.

44 In order to compare some of the indicators presented in this section with those related to the whole of the EU and the EA, Table S15 of Additional file 4 presents data extracted from the use tables at basic prices in the form of indices (EU = 100) in per capita and PPS terms. The difference between the information collected above and that provided in Additional file 4 is a consequence of the methods of valuation of the transactions, since the distribution margins (trade and transport mainly) are not included in the use tables at basic prices nor the corresponding taxes on products.

45 Price level indices express the price level of one country in relation to that of another, or, as in this case, with the price level of a group of countries, such as the European Union. They are calculated as a quotient between PPPs and the nominal exchange rate of the corresponding year. If the referred index is higher than 100, the corresponding country is relatively more expensive than another (or, as in this case, the EU price level), while if it is less than 100, it is relatively cheaper than other countries.

46 If PPPs for GDP are considered, the coefficients of variation increase markedly: 0.41, when the GVA is used, and 0.37 when the output value is used. Again, the use of health specific PPPs generates a homogenizing effect: it reduces labour productivity in health activities in those countries with higher prices for health and increases labour productivity in health activities in those countries that register lower prices for this type of services (see Figure S1 of Additional File 4). As happened with the FCE, the ranking of countries according to apparent labour productivity in health care activities changes considerably depending on the PPP used (21 out of 26 countries change the position in the ranking when the PPP is changed).
