# Supplementary material for: The health care sector in the economies of the European Union: an overview using an input–output framework
Source: Cost Eff Resour Alloc. 2021 Jan 19;19:4. doi: 10.1186/s12962-021-00258-8 (PMC7816493; doi:10.1186/s12962-021-00258-8)
Supplement: Supplementary file 1 — Additional file 1. Methodological summary of input-output analysis (.doc). Additional file 1 contains a methodological summary of the input-output framework and the analysis applied in this study. [file 12962_2021_258_MOESM1_ESM.pdf]

ADDITIONAL FILE 1. Methodological summary of input-output analysis

SYMMETRIC INPUT-OUTPUT TABLE (product-by-product or industry-by-industry)

Table S1. Symmetric input-output table (product by product)

<table>
<tr><td colspan="2" rowspan="2"></td><td colspan="3">Products produced</td><td rowspan="2">Final demand</td><td rowspan="2">Total</td></tr>
<tr><td>Agricultural products</td><td>Industrial products</td><td>Services</td></tr>
<tr><td rowspan="4">Products used</td><td>Agricultural products</td><td colspan="3" rowspan="3">S</td><td rowspan="3">y</td><td rowspan="3">q</td></tr>
<tr><td>Industrial products</td></tr>
<tr><td>Services</td></tr>
<tr><td>Value added</td><td colspan="3">e</td><td colspan="2"></td></tr>
<tr><td></td><td>Total</td><td colspan="3">q^T</td><td colspan="2"></td></tr>
</table>

S = matrix for intermediates (product by product), with $S = s_{ij}$

e = vector of value added (by products)

q = column vector of output (by products); q^T = row vector of product output

y = vector of exogenous final demand (by products)

Model: $q = (I - A)^{-1} y$

I = unit matrix

A = matrix of input (direct) coefficients for intermediates, with $A = a_{ij}$

$a_{ij} = s_{ij}/q_j$ (Input coefficients for products [technical coefficient])

$A = S$ (diag(q))$^{-1}$

$(I - A)^{-1}$ = Leontief inverse or the total (direct and indirect) requirements matrix. The Leontief inverse reflects the dependence of each of the gross outputs on the values of each of the final demands. It is a product-by-product total requirements matrix.

This model is often used to study the impact on gross output (by products or by industries) following a change in exogenous final demand.

Gross output requirements could be translated into employment effects (in physical terms – for example, hours worked), or effects on value-added (in euros).

Value-added multipliers

e = vector of value added (by products)

f = vector of input coefficients for value-added (euros of value-added per unit of output)

$f = e\ [\mathrm{diag}(q)]^{-1}$

$E = f\ (I - A)^{-1}$: vector with results for direct and indirect requirements for value added (by products) per unit of additional final demand

Employment multipliers

t = vector of hours worked (by products)

h = vector of input coefficients for labour (hours worked per unit of output)

$h = t\ [\mathrm{diag}(q)]^{-1}$

$H = h\ (I - A)^{-1}$: vector with results for direct and indirect requirements for labour (by products) per unit of additional final demand.

SUPPLY TABLE

Table S2. Supply table

		Producing industries			Total supply by product
Supplies		Agriculture	Industry	Service activities	
Products	Agricultural products	$\mathbf{V}^T$			**q**
	Industrial products				
	Services				
	Total industry output	$\mathbf{g}^T$			

V = make matrix - transpose of supply matrix (industry by product) [v_{ij}] shows the value of the output of product *j* that is produced by industry *i*.

V^T= supply matrix (product by industry)

$d_{ij} = v_{ij}/q_j$ denotes the fraction of total product *j* output that was produced by industry *i*. Forming a matrix of these product output proportions, *D*, we have

$D = V\,[\text{diag}(q)]^{-1}$: market shares matrix (contribution of each industry to the output of a product)

g = column vector of industry output; g^T = row vector of industry output

USE TABLE

Table S3. Use table

		Industries			Final demand	Total use by product
Uses		Agriculture	Industry	Service activities		
Products	Agricultural products	**U**			**y**	**q**
	Industrial products					
	Services					
	Value added	**w**				
	Output	$\mathbf{g}^T$				

U = use matrix for intermediates (product by industry), where u_{ij} is the value of purchases of product i by industry j. In conjunction with total industry output, g, the parallel to ordinary technical coefficients, a_{ij}, would appear to be $z_{ij} = u_{ij}/g_j$, or

$Z = U\,[\text{diag}(g)]^{-1}$: input requirements for products per unit of output of an industry (intermediates)

w = vector of value added (by industry)

y = vector of exogenous final demand (by products)

Four standard models can be used for the transformation to product-by-product input-output tables or industry-by-industry input-output tables, and to derive total requirements matrices (see Eurostat [1], Miller and Blair [2], and United Nations [3]). The four basic transformation models are based on the following assumptions:

- Product technology assumption (Model A): Each product is produced in its own specific way, irrespective of the industry where it is produced. Negative elements may occur.
- Industry technology assumption (Mode B): Each industry has its own specific way of production, irrespective of its product mix. No negative elements.
- Fixed industry sales structure assumption (Model C): Each industry has its own specific sales structure, irrespective of its product mix. Negative elements may occur.
- Fixed product sales structure assumption (Model D): Each product has its own specific sales structure, irrespective of the industry where it is produced. No negative elements.

The selection of the appropriate type of input-output tables (product by product vs. industry by industry) depends on the specific objective of economic analysis. There is, however, no consensus of which should be preferred.

In this work, we have chosen Model D, as, as expressed in the Eurostat Manual (page 310) [1], "Industry-by-industry tables which are based on the fixed product sales structure (Model D) do not involve any technology assumptions (A and B), and do not require the

application of sometimes arbitrary methods to adjust for negatives (A and C). Furthermore, Table D retains the links to the national accounts data and basic statistics and requires fewer resources to compile. It should also be noted that the overall sales share in a row is not an assumption, but actually observed" (see also Miller and Blair, page 209 [2]). More specifically, we have also chosen the model indicated because the information on employment data (number of hours worked) available in Eurostat refers to the existing one by industries and not by products.

MODEL D: $g = [(I - DZ)^{-1} D]\, y$, or, $g = [D\,(I - ZD)^{-1}]\, y$ (Miller and Blair, page 191 [2])

The inverse on the right-hand side [the bracketed matrix] connects product final demand to industry output. It is an industry-by-product total requirements matrix. It thus plays the role of the Leontief inverse $[(I-A)^{-1}]$ in the ordinary input–output model (symmetric input-output table).

Value-added multipliers

w = vector of value added (by industry)

p = vector of input coefficients for value-added (euros of value-added per unit of industry output)

$p = w\,[\mathrm{diag}(g)]^{-1}$

$W = p\,[D\,(I - ZD)^{-1}]$: vector with results for direct and indirect requirements for value added by industry (euros) per unit of additional final demand (by product)

Employment multipliers

r = vector of hours worked (by industry)

k = vector of input coefficients for labour (hours worked per unit of industry output)

$k = r\,[\mathrm{diag}(g)]^{-1}$

$R = k\,[D\,(I - ZD)^{-1}]$: vector with results for direct and indirect requirements for labour by industry (hours worked) per unit of additional final demand (by product).

REFERENCES

1. EUROSTAT. Eurostat Manual of Supply, Use and Input-Output Tables. Luxembourg2008.

2. MILLER RE, BLAIR PD. Input-Output Analysis: Foundations and Extensions, Second Edition. Cambridge University Press. 2009.

3. UNITED NATIONS. Handbook on Supply, Use and Input-Output Tables with Extensions and Applications. Studies in Methods-Handbook of National Accounting. In: Division S, editor.2018.
