# Supplementary material for: The health care sector in the economies of the European Union: an overview using an input–output framework
Source: Cost Eff Resour Alloc. 2021 Jan 19;19:4. doi: 10.1186/s12962-021-00258-8 (PMC7816493; doi:10.1186/s12962-021-00258-8)
Supplement: Supplementary file 2 — Additional file 2. Sources of information (.doc). Additional file 2 explains in detail the sources of information used in this study [file 12962_2021_258_MOESM2_ESM.pdf]

ADDITIONAL FILE 2. Sources of information

The main source of data used in this paper corresponds to the Input-Output Framework of the different countries of the European Union (EU), as well as for the whole of the EU (28 countries) and the Economic and Monetary Union (EA, 19 countries), published by EUROSTAT in its database [1]. The methodological basis that supports such data is the European System of National and Regional Accounts of the European Union (ESA 2010) [2]. The reference year is 2010, the last year for which, during the preparation of this study, SIOT had been published for a significant number of countries. More specifically, the following tables (current prices) have been downloaded:

- Supply table at basic prices (product by industry) [3]
- Use table at purchasers' prices (product by industry) [4]
- Use table at basic prices (product by industry) [5]
- Symmetric input-output table at basic prices (product by product) [6]

All these tables in their original composition are presented with a disaggregation into 64 types of products or industries[1]. However, the need to complement the aforementioned information with data related to employment made it necessary to proceed with an aggregation of that number of industries to only 37, so that it would be possible to reconcile that information with that on employment (number of hours worked and number of persons employed) also publishes Eurostat in its database [7]. The correspondence table used is presented in Additional file 3.

[1] In fact, the original number of industries/ products was 65, but as the industry/product No. 65 (Activities of organizations and extraterritorial bodies/Services of organizations and extraterritorial organizations) did not record any value in the tables of the different countries, it was decided to omit it from all the calculations made.

As can be seen in the paper, not all tables present information for the same countries or for the same number of countries. Such differences are justified by a variety of situations in the downloaded information:

- No publication of some tables for certain countries. As an example, during the preparation of this document EUROSTAT has not published for the reference year use tables at acquisition prices for the EU or the EA, nor use tables at basic prices for Germany or SIOT for Denmark, the Netherlands, Portugal or Finland.

- Some of the published tables are not complete, as there is a lack of information on certain activity industries or products. Without being exhaustive, and by way of example, this situation is verified in the supply tables of Bulgaria, Estonia, Ireland, Lithuania, Luxembourg, Poland, Finland, Romania or Sweden; in the use tables at purchasers' prices or at basic prices in Estonia, Ireland, Luxembourg, Malta, Poland, Finland or Sweden; in the SIOT of the Czech Republic, Estonia, Ireland, Lithuania or Sweden.

- For some countries, certain tables are not presented for all possible supplies of the exchanges (domestic, imported, total).

- It has been shown that in some tables for certain countries, and although the information appears to be complete, the data recorded are not consistent. Thus, for example, the total output by industries and by products does not match in the supply and use tables of the United Kingdom. In some countries, it was not possible to verify the correct result after the implementation of the corresponding model (exogenous final demand for products that generates a certain output by products or by industries, depending on the case -SIOT or tables of supply and use-) (see Additional file 2 on input-output), as are the cases (supply and use tables) of the Czech Republic, Greece or the United Kingdom.

Owing to the above, only the different multipliers or requirements have been estimated in cases where it has been verified that the information was correct.

In spite of this, in the cases in which the variables related to final consumption expenditure (FCE) of households or general government (GG) in health care services are analysed, it has been decided to use all available information registered in the use tables (at purchasers' prices) of the different countries, after checking that: 1) for the majority of the EU countries, the total FCE of the GG collected in the use tables (purchasers' prices)) coincides, or presents a deviation of less than 1%, with that offered in the statistics (Eurostat) FCE of the GG by functions [Government finance statistics (ESA 2010) -General government expenditure by function (COFOG)], percentage that is only exceeded in the cases of Ireland (2.2%), Cyprus (2.1%), Lithuania (1.1%), Romania (1.4%) and Slovakia (2.8%); 2) for almost all EU countries, the total FCE of households included in the use tables (purchasers' prices) coincides, or shows a deviation of less than 1%, with that offered in the statistics (Eurostat) Household FCE by purpose [Final consumption expenditure of households by consumption purpose (COICOP)], the only exception being the case of Latvia (2.3%). Likewise, in the cases in which the information corresponding to the health care activities (columns) and health care services (rows and/or columns) of the different tables is complete, it has been decided to use the referred information, although for other industries or products there are omitted values.

The rest of the information used, also published by EUROSTAT, corresponds to the following variables:

- Population: Population on 1 January by age and sex [8]
- Purchasing power parities: Purchasing power parities (PPPs), price level indices and real expenditures for ESA 2010 aggregates [9]
- Exchange rates: Euro / ECU exchange rates - annual data [10]

REFERENCES

1. EUROSTAT. Database. 2019. http://ec.europa.eu/eurostat/data/database. Accessed November, 2016 - October, 2017.

2. European system of accounts - ESA 2010. Regulation [EU] nº 549/2013 of the European Parliament and of the Council of 21 May 2013 on the European System of National and Regional Accounts in the European Union, (2013).

3. EUROSTAT. Supply table at basic prices (product by industry) 2019. https://appsso.eurostat.ec.europa.eu/nui/show.do?dataset=naio_10_cp15&lang=en. Accessed November, 2016 - October, 2017.

4. EUROSTAT. Use table at purchasers' prices (product by industry). 2019. https://appsso.eurostat.ec.europa.eu/nui/show.do?dataset=naio_10_cp16&lang=en. Accessed November, 2016 - October, 2017.

5. EUROSTAT. Use table at basic prices (product by industry). 2019. https://appsso.eurostat.ec.europa.eu/nui/show.do?dataset=naio_10_cp1610&lang=en. Accessed November, 2016 - October, 2017.

6. EUROSTAT. Symmetric input-output table at basic prices (product by product). 2019. https://appsso.eurostat.ec.europa.eu/nui/show.do?dataset=naio_10_cp1700&lang=en. Accessed November, 2016 - October, 2017.

7. EUROSTAT. National Accounts employment data by industry (up to NACE A * 64). 2019. https://appsso.eurostat.ec.europa.eu/nui/show.do?dataset=nama_10_a64_e&lang=en. Accessed November, 2016 - October, 2017.

8. EUROSTAT. Population: Population on 1 January by age and sex. 2019. https://appsso.eurostat.ec.europa.eu/nui/show.do?dataset=demo_pjan&lang=en. Accessed November, 2016 - October, 2017.

9. EUROSTAT. Purchasing power parities: Purchasing power parities (PPPs), price level indices and real expenditures for ESA 2010 aggregates. 2019. https://appsso.eurostat.ec.europa.eu/nui/show.do?dataset=prc_ppp_ind&lang=en. Accessed November, 2016 - October, 2017.

10. EUROSTAT. Exchange rates: Euro / ECU exchange rates - annual data. 2019. https://appsso.eurostat.ec.europa.eu/nui/show.do?dataset=ert_bil_eur_a&lang=en. Accessed November, 2016 - October, 2017.
