# Supplementary material for: The health care sector in the economies of the European Union: an overview using an input–output framework
Source: Cost Eff Resour Alloc. 2021 Jan 19;19:4. doi: 10.1186/s12962-021-00258-8 (PMC7816493; doi:10.1186/s12962-021-00258-8)
Supplement: Supplementary file 3 — Additional file 3. Correspondence table of industries/products with the Eurostat input-output framework and the NACE rev. 2/CPA 2008 (.doc). Additional file 3 contains a table with industries/products and their respective Eurostat input-output framework and the NACE rev. 2/CPA 2008. [file 12962_2021_258_MOESM3_ESM.pdf]

ADDITIONAL FILE 3.

Table S4. Correspondence table of industries/products with the Eurostat input-output framework and the NACE rev. 2/CPA 2008

Aggregations of industries/ products	INDUSTRIES	PRODUCTS	INDUSTRIES/ PRODUCTS EUROSTAT TABLES	CPA 2008 / NACE rev. 2
**1**	Agriculture, forestry and fishing	Products of agriculture, forestry and fishing	**1 a 3**	**01-03**
**2**	Mining and quarrying	Mining and quarrying	**4**	**05-09**
**3**	Manufacture of food products; beverages and tobacco products	Food, beverages and tobacco products	**5**	**10-12**
**4**	Manufacture of textiles, wearing apparel, leather and related products	Textiles, wearing apparel, leather and related products	**6**	**13-15**
**5**	Manufacture of wood, paper, printing and reproduction	Wood and of products of wood and cork, except furniture; articles of straw and plaiting materialspaper and paper products; printing and recording services	**7 a 9**	**16-18**
**6**	Manufacture of coke and refined petroleum products	Coke and refined petroleum products	**10**	**19**
**7**	Manufacture of chemicals and chemical products	Chemicals and chemical products	**11**	**20**
**8**	Manufacture of basic pharmaceutical products and pharmaceutical preparations	Basic pharmaceutical products and pharmaceutical preparations	**12**	**21**
**9**	Manufacture of rubber and plastic products and other non-metallic mineral products	Rubber and plastics products; other non-metallic mineral products	**13 y 14**	**22-23**
**10**	Manufacture of basic metals and fabricated metal products, except machinery and equipment	Basic metals; fabricated metal products, except machinery and equipment	**15 y 16**	**24-25**
**11**	Manufacture of computer, electronic and optical products	Computer, electronic and optical products	**17**	**26**
**12**	Manufacture of electrical equipment	Electrical equipment	**18**	**27**
**13**	Manufacture of machinery and equipment n.e.c.	Machinery and equipment n.e.c.	**19**	**28**

Aggregations of industries/ products	INDUSTRIES	PRODUCTS	INDUSTRIES/ PRODUCTS EUROSTAT TABLES	CPA 2008 / NACE rev. 2
**14**	Manufacture of motor vehicles, trailers, semi-trailers and of other transport equipment	Motor vehicles, trailers and semi-trailers; other transport equipment	**20 y 21**	**29-30**
**15**	Manufacture of furniture; jewellery, musical instruments, toys; repair and installation of machinery and equipment	Furniture; other manufactured goods repair and installation services of machinery and equipment	**22 y 23**	**31-33**
**16**	Electricity, gas, steam and air conditioning supply	Electricity, gas, steam and air conditioning	**24**	**35**
**17**	Water supply; sewerage, waste management and remediation activities	Water supply; sewerage, waste management and remediation services	**25 y 26**	**36-39**
**18**	Construction	Constructions and construction works	**27**	**41-43**
**19**	Wholesale and retail trade; repair of motor vehicles and motorcycles	Wholesale and retail trade services; repair services of motor vehicles and motorcycles	**28 a 30**	**45-47**
**20**	Transportation and storage	Transportation and storage services	**31 a 35**	**49-53**
**21**	Accommodation and food service activities	Accommodation and food services	**36**	**55-56**
**22**	Publishing, motion picture, video, television programme production; sound recording, programming and broadcasting activities	Publishing services; motion picture, video and television programme production services, sound recording and music publishing; programming and broadcasting services	**37 y 38**	**58-60**
**23**	Telecommunications	Telecommunications services	**39**	**61**
**24**	Computer programming, consultancy, and information service activities	Computer programming, consultancy and related services; information services	**40**	**62-63**
**25**	Financial and insurance activities	Financial and insurance services	**41 a 43**	**64-66**
**26**	Real estate activities	Real estate services	**44 y 44a**	**68**
**27**	Legal and accounting activities; activities of head offices; management consultancy activities; architectural and engineering activities; technical testing and analysis	Legal and accounting services; services of head offices; management consulting services; architectural and engineering services; technical testing and analysis services	**45 y 46**	**69-71**
**28**	Scientific research and development	Scientific research and development services	**47**	**72**

Aggregations of industries/ products	INDUSTRIES	PRODUCTS	INDUSTRIES/ PRODUCTS EUROSTAT TABLES	CPA 2008 / NACE rev. 2
**29**	Advertising and market research; other professional, scientific and technical activities; veterinary activities	Advertising and market research services; other professional, scientific and technical services; veterinary services	**48 y 49**	**73-75**
**30**	Administrative and support service activities	Administrative and support services	**50 a 53**	**77-82**
**31**	Public administration and defence; compulsory social security	Public administration and defence services; compulsory social security services	**54**	**84**
**32**	Education	Education services	**55**	**85**
**33**	Human health activities	Human health services	**56**	**86**
**34**	Residential care activities and social work activities without accommodation	Residential care services; social work services without accommodation	**57**	**87-88**
**35**	Arts, entertainment and recreation	Arts, entertainment and recreation services	**58 y 59**	**90-93**
**36**	Other service activities	Other services	**60 a 62**	**94-96**
**37**	Activities of households as employers; undifferentiated goods- and services-producing activities of households for own use	Services of households as employers; undifferentiated goods and services produced by households for own use	**63**	**97-98**
	Activities of extraterritorial organisations and bodies	Services provided by extraterritorial organisations and bodies	**64**	**99**

[a] The aggregation of industries is the same used for the data of hours worked and persons employed published by EUROSTAT on the basis of the same classification (NACE rev. 2).
