# Supplementary material for: The health care sector in the economies of the European Union: an overview using an input–output framework
Source: Cost Eff Resour Alloc. 2021 Jan 19;19:4. doi: 10.1186/s12962-021-00258-8 (PMC7816493; doi:10.1186/s12962-021-00258-8)
Supplement: Supplementary file 4 — Additional file 4. Complementary tables and figures (.doc). Additional file 4 contains eleven tables with complementary information on health care services, health care activities, direct and indirect requirements for GVA and for hours worked. And it also contains one figure with a comparison of apparent labour productivity on health care activities using PPPs for GDP and for health. [file 12962_2021_258_MOESM4_ESM.pdf]

ADDITIONAL FILE 4. Complementary tables and figures

Table S5. Health care activities (use table). Direct backward linkages (%) (domestic, basic prices). 2010

Products	EU (28)	EA (19)	Belgium	Denmark	Spain	France	Croatia	Italy	Cyprus	Hungary	Netherlands	Austria	Poland	Portugal	Slovenia
**1**	0.06	0.06	0.06	0.01	0.07	0.00	0.05	0.01	0.51	0.06	0.09	0.01	0.09	0.01	0.01
**2**	0.02	0.02	0.00	0.05	0.00	0.01	0.01	0.01	0.00	0.08	0.18	0.00	0.01	0.00	0.10
**3**	0.94	0.97	0.39	0.18	0.65	1.00	0.22	0.16	3.65	0.48	0.58	0.38	0.27	0.07	0.33
**4**	0.14	0.10	0.09	0.02	0.12	0.11	0.00	0.05	0.41	0.03	0.08	0.05	0.03	0.10	0.00
**5**	0.32	0.18	0.20	0.03	0.13	0.10	0.11	0.10	0.14	0.09	0.49	0.06	0.16	0.26	0.01
**6**	0.11	0.11	0.24	0.02	0.04	0.06	0.15	0.07	0.00	0.15	0.01	0.17	0.24	0.22	0.00
**7**	0.59	0.58	0.65	0.05	1.20	0.17	0.07	0.78	0.08	0.72	0.14	0.06	0.63	1.88	0.00
**8**	2.83	2.39	3.67	1.79	2.12	0.44	8.62	1.74	0.14	2.03	0.36	1.21	1.46	1.78	1.46
**9**	0.25	0.21	0.04	0.07	0.24	0.25	0.07	0.07	0.37	0.05	0.06	0.18	0.15	0.05	0.00
**10**	0.06	0.05	0.04	0.03	0.01	0.08	0.01	0.02	0.14	0.03	0.04	0.01	0.04	0.05	0.03
**11**	0.49	0.36	0.01	0.08	0.14	0.13	0.01	0.26	0.02	0.04	0.02	0.05	0.08	0.00	0.00
**12**	0.03	0.02	0.00	0.01	0.00	0.00	0.01	0.00	0.02	0.00	0.05	0.00	0.10	0.00	0.00
**13**	0.03	0.02	0.02	0.02	0.00	0.01	0.02	0.01	0.00	0.00	0.00	0.00	0.00	0.00	0.03
**14**	0.04	0.02	0.01	0.00	0.00	0.03	0.00	0.01	0.02	0.00	0.00	0.00	0.01	0.00	0.04
**15**	1.33	1.34	0.55	1.15	2.12	0.76	6.28	1.65	0.16	0.71	0.12	1.16	1.49	1.05	1.34
**16**	0.92	0.86	0.43	0.57	0.92	0.44	0.96	0.85	2.32	1.54	0.31	1.23	3.62	1.04	0.77
**17**	0.52	0.38	0.17	1.09	0.17	0.44	1.00	0.23	0.69	0.57	0.55	0.34	0.30	0.44	0.33
**18**	0.75	0.81	0.32	0.01	0.42	0.17	0.39	1.08	0.75	0.30	0.58	1.16	1.00	0.89	0.62
**19**	4.03	4.27	3.94	2.04	5.52	2.59	6.21	2.12	3.76	3.15	3.33	3.27	3.49	4.23	4.23
**20**	0.67	0.48	0.73	1.75	0.27	0.60	0.77	0.40	0.34	0.72	0.92	0.46	0.41	0.38	0.37
**21**	0.54	0.57	0.75	0.11	0.83	0.24	0.00	1.10	0.43	0.54	1.32	0.16	0.15	0.87	0.14

Products	EU (28)	EA (19)	Belgium	Denmark	Spain	France	Croatia	Italy	Cyprus	Hungary	Netherlands	Austria	Poland	Portugal	Slovenia
**22**	0.23	0.27	0.09	0.11	0.18	0.17	0.00	0.05	0.22	0.11	0.40	0.09	0.27	0.04	0.08
**23**	0.37	0.33	0.30	0.47	0.44	0.32	0.16	0.27	0.21	0.53	0.63	0.37	0.82	0.76	0.17
**24**	0.56	0.45	0.89	1.45	0.24	0.16	0.09	0.37	0.00	0.56	0.55	0.54	0.25	0.60	0.36
**25**	1.14	1.20	1.21	0.27	1.08	1.51	0.09	1.09	1.21	0.64	1.10	0.82	0.82	0.66	0.42
**26**	1.27	1.19	1.29	0.93	0.36	0.78	0.01	0.93	2.13	2.27	0.97	2.25	2.02	0.77	0.39
**27**	1.56	1.48	2.08	0.41	0.41	1.05	0.07	3.61	0.33	1.04	1.04	2.35	0.93	3.61	0.93
**28**	0.25	0.06	0.00	0.69	0.00	0.00	0.00	0.23	0.00	0.00	0.00	0.00	0.00	0.00	0.00
**29**	0.35	0.35	0.04	0.10	0.02	0.40	0.16	0.94	0.12	0.04	0.19	0.39	0.57	0.67	0.64
**30**	2.22	2.19	3.46	2.76	2.46	1.76	0.14	2.43	1.00	2.36	2.32	1.58	0.94	2.99	1.49
**31**	0.15	0.14	0.04	1.63	0.03	0.00	0.01	0.01	0.12	0.11	0.14	0.02	0.15	0.00	0.19
**32**	0.32	0.38	0.07	0.03	0.19	1.04	0.08	0.18	0.09	0.04	0.49	0.16	0.06	0.03	0.36
**33**	4.90	4.46	13.35	1.14	5.56	1.19	0.59	6.49	0.21	4.79	3.02	1.88	7.97	4.11	3.84
**34**	0.35	0.22	0.00	0.00	0.01	0.00	0.00	1.06	0.50	0.00	0.26	0.00	0.00	0.00	0.01
**35**	0.02	0.02	0.20	0.01	0.00	0.02	0.00	0.00	0.02	0.02	0.03	0.00	0.02	0.00	0.00
**36**	0.63	0.63	0.99	0.64	0.35	0.04	0.13	1.14	0.40	0.74	0.79	0.46	0.58	0.22	0.50
**37**	0.02	0.00	0.00	0.89	0.00	0.00	0.00	0.00	0.00	0.00	0.00	0.00	0.00	0.00	0.00
**TOTAL**	**29.01**	**27.16**	**36.28**	**20.62**	**26.32**	**16.05**	**26.51**	**29.52**	**20.54**	**24.56**	**21.16**	**20.86**	**29.13**	**27.77**	**19.19**
**Average industries**	**45.45**	**43.61**	**35.69**	**34.43**	**40.40**	**37.90**	**34.23**	**41.55**	**32.12**	**28.62**	**35.33**	**34.62**	**38.95**	**36.52**	**33.76**
**Average service industries**	**36.97**	**35.39**	**33.46**	**30.67**	**33.07**	**31.72**	**30.14**	**35.76**	**26.27**	**27.83**	**32.15**	**32.34**	**34.49**	**33.44**	**29.89**
**Average industries (excl. ind. 37)**	**46.72**	**44.82**	**36.68**	**35.38**	**41.53**	**38.95**	**34.35**	**42.71**	**33.01**	**29.42**	**36.31**	**35.58**	**40.03**	**37.53**	**34.69**
**Average service industries (excl. ind. 37)**	**39.03**	**37.36**	**35.32**	**32.37**	**34.91**	**33.48**	**30.16**	**37.75**	**27.73**	**29.37**	**33.94**	**34.13**	**36.41**	**35.29**	**31.55**

Source: Prepared by authors with Eurostat data, "Use table at basic prices (domestic)”.

Table S6. Health care services (SIOT). Direct backward linkages (%) (domestic, basic prices). 2010

Products	EU (28)	EA (19)	Belgium	Spain	France	Germany	Italy	Hungary	Austria	Greece	UK
**1**	0.06	0.06	0.03	0.07	0.00	0.09	0.02	0.03	0.01	0.02	0.06
**2**	0.02	0.02	0.00	0.00	0.01	0.00	0.01	0.00	0.00	0.02	0.08
**3**	0.94	0.98	0.29	0.66	1.00	1.65	0.16	0.95	0.33	0.59	0.47
**4**	0.14	0.10	0.09	0.12	0.11	0.05	0.05	0.10	0.05	0.04	0.03
**5**	0.32	0.18	0.21	0.12	0.10	0.10	0.10	0.02	0.04	0.53	0.09
**6**	0.11	0.11	0.23	0.04	0.06	0.07	0.07	0.62	0.17	0.64	0.14
**7**	0.58	0.58	0.67	1.20	0.17	0.11	0.78	0.14	0.06	0.48	0.75
**8**	2.81	2.37	3.83	1.96	0.43	0.84	1.73	0.26	1.25	1.51	2.06
**9**	0.25	0.21	0.04	0.24	0.25	0.11	0.07	0.56	0.18	0.63	0.05
**10**	0.06	0.05	0.04	0.01	0.08	0.06	0.02	0.01	0.01	0.09	0.03
**11**	0.48	0.35	0.01	0.12	0.13	0.06	0.26	0.01	0.05	0.14	0.04
**12**	0.03	0.02	0.00	0.00	0.00	0.01	0.00	0.01	0.00	0.03	0.00
**13**	0.03	0.02	0.02	0.00	0.01	0.03	0.01	0.00	0.00	0.05	0.00
**14**	0.04	0.02	0.01	0.00	0.03	0.00	0.01	0.00	0.00	0.12	0.00
**15**	1.33	1.33	0.56	2.12	0.74	0.79	1.64	0.63	1.20	1.69	0.73
**16**	0.92	0.86	0.36	0.92	0.44	1.20	0.85	1.04	1.23	0.64	1.56
**17**	0.52	0.38	0.17	0.17	0.44	0.44	0.23	0.66	0.34	0.53	0.57
**18**	0.75	0.82	0.31	0.42	0.19	1.38	1.07	1.10	1.13	0.17	0.30
**19**	4.02	4.25	4.01	5.50	2.54	5.66	2.12	8.51	3.31	1.97	3.10
**20**	0.68	0.49	0.77	0.27	0.62	0.18	0.41	0.11	0.45	1.23	0.72
**21**	0.55	0.57	0.77	0.83	0.24	0.02	1.10	0.14	0.15	1.02	0.52
**22**	0.23	0.27	0.09	0.18	0.17	0.55	0.05	0.12	0.09	0.20	0.11
**23**	0.37	0.33	0.29	0.44	0.32	0.19	0.27	0.34	0.37	0.82	0.52
**24**	0.56	0.45	0.76	0.24	0.16	0.51	0.38	0.00	0.46	0.94	0.54

Products	EU (28)	EA (19)	Belgium	Spain	France	Germany	Italy	Hungary	Austria	Greece	UK
**25**	1.15	1.20	1.21	1.09	1.49	1.05	1.10	0.38	0.78	4.65	0.63
**26**	1.28	1.20	1.29	0.36	0.80	1.90	0.95	0.58	2.13	5.26	2.24
**27**	1.57	1.49	2.08	0.41	1.07	0.65	3.61	0.86	2.36	4.96	1.02
**28**	0.25	0.06	0.00	0.00	0.00	0.00	0.23	0.13	0.00	0.02	0.00
**29**	0.35	0.35	0.05	0.02	0.40	0.12	0.94	0.17	0.38	1.05	0.04
**30**	2.22	2.19	3.56	2.46	1.77	1.91	2.43	0.43	1.52	3.86	2.33
**31**	0.16	0.14	0.04	0.03	0.00	0.28	0.01	0.00	0.02	0.00	0.11
**32**	0.32	0.39	0.07	0.19	1.02	0.08	0.18	0.46	0.16	0.05	0.04
**33**	4.86	4.43	14.20	5.58	1.20	3.68	6.47	0.84	1.94	1.31	4.79
**34**	0.36	0.22	0.00	0.01	0.00	0.00	1.06	0.00	0.00	0.01	0.00
**35**	0.02	0.02	0.21	0.00	0.03	0.03	0.00	0.00	0.00	0.11	0.02
**36**	0.63	0.63	1.01	0.35	0.04	0.73	1.14	0.84	0.46	0.69	0.72
**37**	0.02	0.00	0.00	0.00	0.00	0.00	0.00	0.00	0.00	0.00	0.00
**TOTAL**	29.03	27.15	37.29	26.15	16.07	24.55	29.54	20.04	20.64	36.09	24.41
**Average industries**	**45.56**	**43.72**	**35.50**	**40.88**	**37.60**	**38.23**	**42.01**	**35.97**	**34.36**	**40.38**	**28.61**
**Average service industries**	**37.27**	**35.70**	**33.34**	**32.64**	**31.59**	**33.90**	**36.42**	**30.92**	**31.20**	**34.61**	**27.76**
**Average industries (excl. ind. 37)**	**46.80**	**44.93**	**36.49**	**42.02**	**38.65**	**39.29**	**43.18**	**36.97**	**35.31**	**41.50**	**29.41**
**Average service industries (excl. ind. 37)**	**39.30**	**37.69**	**35.19**	**34.45**	**33.34**	**35.79**	**38.44**	**32.64**	**32.93**	**36.53**	**29.31**

Source: Prepared by authors with Eurostat data, "Symmetric input-output table at basic prices (product by product) (domestic)".

Table S7. Health care activities (use table). Intermediate consumption by products (% of total intermediate consumption by industry (domestic, basic prices). 2010

Products	EU (28)	EA (19)	Belgium	Denmark	Spain	France	Croatia	Italy	Cyprus	Hungary	Netherlands	Austria	Poland	Portugal	Slovenia
**1**	0.21	0.22	0.16	0.07	0.27	0.00	0.18	0.05	2.48	0.23	0.43	0.04	0.30	0.02	0.04
**2**	0.05	0.06	0.00	0.26	0.00	0.06	0.03	0.03	0.00	0.34	0.84	0.01	0.03	0.00	0.54
**3**	3.24	3.58	1.08	0.85	2.47	6.24	0.84	0.55	17.78	1.96	2.72	1.82	0.92	0.26	1.74
**4**	0.49	0.39	0.24	0.10	0.45	0.67	0.00	0.17	2.02	0.11	0.37	0.24	0.11	0.37	0.01
**5**	1.11	0.66	0.54	0.14	0.50	0.61	0.43	0.35	0.70	0.38	2.31	0.27	0.54	0.92	0.06
**6**	0.38	0.39	0.65	0.12	0.16	0.37	0.57	0.22	0.02	0.62	0.06	0.84	0.84	0.78	0.00
**7**	2.02	2.13	1.78	0.24	4.57	1.05	0.25	2.64	0.41	2.92	0.65	0.31	2.16	6.76	0.00
**8**	9.76	8.79	10.13	8.70	8.06	2.71	32.51	5.88	0.66	8.28	1.72	5.78	5.01	6.41	7.63
**9**	0.87	0.76	0.12	0.36	0.90	1.56	0.28	0.23	1.82	0.19	0.29	0.85	0.52	0.18	0.01
**10**	0.20	0.20	0.11	0.17	0.05	0.48	0.03	0.07	0.70	0.13	0.18	0.04	0.13	0.19	0.15
**11**	1.67	1.31	0.03	0.37	0.54	0.84	0.04	0.89	0.10	0.15	0.12	0.23	0.28	0.00	0.00
**12**	0.09	0.08	0.01	0.02	0.01	0.03	0.03	0.01	0.09	0.02	0.23	0.00	0.36	0.00	0.00
**13**	0.10	0.09	0.06	0.08	0.01	0.08	0.06	0.02	0.02	0.00	0.00	0.00	0.02	0.00	0.14
**14**	0.14	0.08	0.01	0.00	0.02	0.17	0.00	0.04	0.09	0.02	0.00	0.00	0.03	0.00	0.21
**15**	4.59	4.94	1.50	5.59	8.07	4.72	23.68	5.57	0.80	2.88	0.58	5.56	5.12	3.78	6.96
**16**	3.17	3.18	1.20	2.75	3.49	2.73	3.62	2.87	11.28	6.26	1.45	5.89	12.44	3.73	4.01
**17**	1.80	1.38	0.46	5.28	0.63	2.72	3.77	0.79	3.35	2.32	2.61	1.62	1.02	1.58	1.71
**18**	2.58	2.99	0.87	0.03	1.60	1.06	1.46	3.64	3.67	1.21	2.76	5.58	3.45	3.22	3.21
**19**	13.90	15.72	10.85	9.89	20.97	16.12	23.44	7.19	18.32	12.83	15.73	15.65	11.97	15.24	22.02
**20**	2.30	1.77	2.01	8.50	1.01	3.74	2.91	1.36	1.68	2.93	4.35	2.20	1.41	1.38	1.93
**21**	1.87	2.08	2.07	0.53	3.15	1.47	0.00	3.71	2.12	2.22	6.24	0.76	0.51	3.13	0.71
**22**	0.79	0.98	0.24	0.55	0.68	1.06	0.01	0.16	1.08	0.45	1.90	0.42	0.93	0.14	0.41
**23**	1.27	1.22	0.82	2.29	1.66	1.97	0.60	0.93	1.02	2.18	2.98	1.76	2.81	2.73	0.88

Products	EU (28)	EA (19)	Belgium	Denmark	Spain	France	Croatia	Italy	Cyprus	Hungary	Netherlands	Austria	Poland	Portugal	Slovenia
**24**	1.92	1.65	2.45	7.01	0.93	0.99	0.35	1.27	0.02	2.29	2.61	2.59	0.86	2.15	1.89
**25**	3.94	4.40	3.33	1.32	4.11	9.39	0.35	3.70	5.88	2.62	5.20	3.91	2.80	2.38	2.20
**26**	4.38	4.40	3.55	4.53	1.38	4.87	0.04	3.15	10.38	9.25	4.59	10.79	6.92	2.76	2.03
**27**	5.39	5.46	5.73	1.99	1.57	6.54	0.26	12.22	1.62	4.22	4.91	11.28	3.19	13.01	4.83
**28**	0.87	0.21	0.00	3.35	0.00	0.00	0.00	0.78	0.00	0.00	0.00	0.00	0.00	0.00	0.00
**29**	1.21	1.30	0.12	0.46	0.08	2.51	0.61	3.18	0.57	0.16	0.90	1.87	1.96	2.41	3.36
**30**	7.64	8.05	9.54	13.41	9.35	10.99	0.54	8.24	4.86	9.63	10.97	7.59	3.24	10.77	7.75
**31**	0.53	0.52	0.10	7.90	0.10	0.00	0.05	0.02	0.57	0.44	0.67	0.10	0.51	0.00	0.99
**32**	1.10	1.41	0.18	0.14	0.72	6.46	0.30	0.62	0.43	0.17	2.31	0.79	0.21	0.10	1.87
**33**	16.87	16.41	36.79	5.53	21.12	7.40	2.24	21.98	1.00	19.49	14.25	9.01	27.36	14.79	20.03
**34**	1.21	0.81	0.00	0.00	0.05	0.00	0.00	3.60	2.46	0.00	1.21	0.00	0.00	0.00	0.03
**35**	0.07	0.07	0.54	0.05	0.01	0.15	0.01	0.01	0.10	0.08	0.14	0.00	0.08	0.02	0.02
**36**	2.19	2.32	2.72	3.13	1.32	0.26	0.50	3.86	1.94	3.02	3.75	2.22	1.98	0.78	2.61
**37**	0.06	0.00	0.00	4.31	0.00	0.00	0.00	0.00	0.00	0.00	0.00	0.00	0.00	0.00	0.00
**TOTAL**	**100.00**	**100.00**	**100.00**	**100.00**	**100.00**	**100.00**	**100.00**	**100.00**	**100.00**	**100.00**	**100.00**	**100.00**	**100.00**	**100.00**	**100.00**

Source: Prepared by authors with Eurostat data, "Use table at basic prices (domestic)".

Table S8. Health care activities (use table). Intermediate consumption by products (% of total intermediate consumption by industry (domestic and imports, basic prices). 2010

Products	EU (28)	EA (19)	Belgium	Denmark	Spain	France	Italy	Hungary	Netherlands	Austria	Poland	Portugal
**1**	0.23	0.26	0.15	0.17	0.26	0.00	0.04	0.20	0.53	0.09	0.31	0.03
**2**	0.12	0.15	0.01	0.21	0.00	0.06	0.19	1.50	0.79	0.05	0.03	0.00
**3**	3.06	3.50	1.22	1.10	2.23	5.55	0.47	1.73	2.66	2.21	0.90	0.25
**4**	0.68	0.56	0.32	0.47	0.45	1.38	0.15	0.18	1.02	0.70	0.16	0.44
**5**	1.04	0.67	1.03	0.43	0.50	0.48	0.30	0.27	2.95	0.71	0.53	0.78
**6**	0.41	0.45	0.69	0.17	0.27	0.43	0.19	0.59	0.07	2.83	0.85	0.79
**7**	2.08	2.28	2.45	0.84	5.47	1.94	2.31	4.80	2.79	1.92	5.38	8.91
**8**	13.07	12.38	16.26	16.93	16.60	12.16	13.95	23.50	11.64	16.19	13.82	20.42
**9**	0.86	0.82	0.20	2.51	0.88	1.70	0.21	0.52	1.80	2.91	0.64	0.28
**10**	0.19	0.20	0.16	0.26	0.05	0.57	0.07	0.31	0.13	0.38	0.21	0.23
**11**	3.53	1.83	0.16	0.12	0.86	2.71	1.17	3.40	3.05	0.52	2.12	0.38
**12**	0.10	0.10	0.02	0.10	0.02	0.08	0.01	0.07	0.24	0.15	0.86	0.01
**13**	0.12	0.13	0.16	0.21	0.02	0.27	0.02	0.21	0.07	0.00	0.14	0.03
**14**	0.15	0.09	0.11	0.01	0.03	0.27	0.04	0.02	0.00	0.00	0.05	0.00
**15**	5.45	6.59	3.56	8.82	8.98	9.13	7.21	10.54	4.31	9.31	7.36	5.31
**16**	2.88	2.89	1.13	2.16	2.91	2.06	2.46	4.14	1.10	4.26	9.88	2.75
**17**	1.72	1.26	0.39	4.09	0.55	2.03	0.68	1.45	1.91	1.17	0.81	1.17
**18**	2.32	2.69	0.72	0.02	1.34	0.78	3.12	0.76	2.01	4.05	2.74	2.39
**19**	12.57	14.27	9.09	7.58	17.64	12.63	6.16	8.03	11.45	11.34	9.51	11.27
**20**	2.14	1.69	2.02	6.85	0.87	3.38	1.27	1.96	3.45	1.94	0.84	1.24
**21**	1.73	1.93	1.81	0.40	2.80	1.08	3.27	1.42	4.59	0.94	0.41	2.52
**22**	0.73	0.92	0.29	0.51	0.62	0.86	0.14	0.28	1.47	0.49	0.83	0.12
**23**	1.18	1.16	0.93	1.91	1.46	1.52	0.95	1.53	2.33	1.28	2.23	2.26

Products	EU (28)	EA (19)	Belgium	Denmark	Spain	France	Italy	Hungary	Netherlands	Austria	Poland	Portugal
**24**	1.80	1.59	2.31	6.23	0.96	0.75	1.23	1.44	2.13	1.91	0.69	1.77
**25**	3.61	4.08	3.18	1.07	3.57	7.20	3.43	1.73	4.28	2.93	2.26	1.94
**26**	3.96	3.96	2.92	3.44	1.15	3.60	2.72	5.79	3.34	7.81	5.50	2.04
**27**	5.02	5.08	6.12	1.75	1.38	5.20	10.83	2.65	4.60	8.21	2.57	10.04
**28**	0.85	0.25	0.00	3.78	0.00	0.00	0.75	0.00	0.00	0.00	0.00	0.00
**29**	1.16	1.25	0.14	0.40	0.07	2.02	3.08	0.10	0.72	1.36	1.57	2.48
**30**	7.14	7.52	9.23	10.97	8.57	9.58	7.38	6.27	8.21	5.51	2.58	8.58
**31**	0.48	0.47	0.08	6.06	0.08	0.00	0.02	0.28	0.49	0.07	0.41	0.00
**32**	1.00	1.28	0.15	0.16	0.60	4.78	0.53	0.11	1.77	0.58	0.17	0.08
**33**	15.38	14.82	30.32	4.59	17.61	5.50	19.07	12.27	10.38	6.55	21.73	10.92
**34**	1.09	0.73	0.00	0.00	0.04	0.00	3.08	0.00	0.88	0.00	0.00	0.00
**35**	0.07	0.06	0.45	0.05	0.01	0.11	0.01	0.05	0.10	0.00	0.06	0.01
**36**	2.00	2.10	2.25	2.37	1.15	0.19	3.49	1.89	2.73	1.61	1.57	0.58
**37**	0.05	0.00	0.00	3.27	0.00	0.00	0.00	0.00	0.00	0.00	0.00	0.00
**TOTAL**	**100**	**100**	**100**	**100**	**100**	**100**	**100**	**100**	**100**	**100**	**100**	**100**

Source: Prepared by authors with Eurostat data, "Use table at basic prices (domestic and imports)”.

Table S9. Health care activities (use table). Intermediate consumption by products (% of total intermediate consumption by industry) (domestic and imports, purchasers' prices). 2010

Products	Belgium	Denmark	Spain	France	Italy	Hungary	Netherlands	Austria	Poland	Portugal	Germany
**1**	0.17	0.22	0.38	0.00	0.05	0.24	0.78	0.11	0.39	0.04	0.84
**2**	0.01	0.36	0.00	0.07	0.43	2.01	1.26	0.07	0.03	0.00	0.44
**3**	1.55	1.31	2.73	7.20	0.53	2.15	3.28	2.70	1.20	0.37	9.91
**4**	0.51	0.55	0.70	1.86	0.19	0.24	1.45	0.90	0.19	0.62	0.62
**5**	1.44	0.49	0.60	0.63	0.35	0.32	3.80	0.92	0.59	0.89	0.75
**6**	1.33	0.37	0.71	0.74	0.35	1.22	0.21	5.15	1.63	1.69	0.67
**7**	3.89	1.24	8.14	2.23	2.75	5.89	3.87	2.62	6.23	10.95	1.96
**8**	20.61	21.66	23.86	15.43	17.64	26.93	15.83	20.30	18.47	26.60	16.31
**9**	0.29	3.61	1.10	2.45	0.26	0.65	2.43	3.53	0.78	0.37	0.69
**10**	0.20	0.29	0.06	0.81	0.10	0.40	0.19	0.47	0.25	0.35	0.25
**11**	0.35	0.15	1.55	4.54	1.65	3.59	4.64	0.66	2.55	0.54	0.55
**12**	0.03	0.15	0.02	0.11	0.01	0.08	0.37	0.21	1.02	0.01	0.12
**13**	0.38	0.28	0.04	0.42	0.03	0.31	0.13	0.00	0.17	0.04	0.66
**14**	0.14	0.01	0.04	0.32	0.06	0.05	0.00	0.00	0.07	0.00	0.01
**15**	4.65	10.35	13.69	13.45	8.82	12.79	5.83	12.23	9.59	6.29	12.58
**16**	1.28	3.86	3.23	2.21	2.95	4.14	1.43	4.61	11.10	2.59	4.63
**17**	0.39	4.23	0.58	2.03	0.71	1.54	2.02	1.13	0.77	1.08	1.43
**18**	0.75	0.02	1.37	0.84	3.01	0.79	2.18	4.13	2.65	2.55	5.11
**19**	1.24	0.06	0.20	0.54	0.26	0.30	0.67	0.77	0.13	0.35	0.42
**20**	1.91	5.54	0.72	2.32	0.47	1.32	2.42	1.43	0.85	1.19	0.66
**21**	1.80	0.41	2.78	1.04	3.10	1.47	4.47	0.90	0.40	2.51	0.20
**22**	0.36	0.63	0.92	0.92	0.18	0.30	1.50	0.60	1.06	0.17	2.58
**23**	1.02	1.95	1.57	1.62	1.09	1.73	2.51	1.30	2.29	2.41	0.74

Products	Belgium	Denmark	Spain	France	Italy	Hungary	Netherlands	Austria	Poland	Portugal	Germany
**24**	2.54	6.41	1.01	0.79	1.31	1.55	2.30	1.94	0.71	1.88	2.40
**25**	3.05	0.90	3.53	6.90	3.26	1.61	3.90	2.68	2.06	1.82	3.72
**26**	2.79	2.95	1.21	3.57	2.82	5.60	3.04	7.40	5.37	1.82	0.00
**27**	6.45	1.83	1.61	5.72	11.84	2.78	4.86	8.32	2.61	10.64	2.52
**28**	0.00	3.89	0.00	0.00	0.81	0.00	0.00	0.00	0.00	0.00	0.00
**29**	0.15	0.41	0.07	2.05	3.20	0.10	0.78	1.39	1.60	2.65	0.52
**30**	10.18	11.24	9.20	9.49	7.74	6.28	8.84	5.59	2.96	9.14	7.20
**31**	0.07	5.64	0.08	0.00	0.01	0.25	0.44	0.06	0.37	0.00	0.88
**32**	0.17	0.13	0.56	4.50	0.52	0.10	1.61	0.59	0.15	0.07	0.28
**33**	27.64	3.81	16.46	4.90	17.20	11.28	9.40	5.68	19.80	9.75	11.51
**34**	0.00	0.00	0.04	0.00	2.97	0.00	0.80	0.00	0.00	0.00	0.00
**35**	0.44	0.04	0.01	0.11	0.01	0.05	0.10	0.00	0.07	0.01	0.12
**36**	2.21	2.31	1.22	0.20	3.33	1.95	2.62	1.61	1.64	0.59	2.51
**37**	0.00	2.71	0.00	0.00	0.00	0.00	0.00	0.00	0.00	0.00	0.00
**TOTAL**	**100**	**100**	**100**	**100**	**100**	**100**	**100**	**100**	**100**	**100**	**100**

Source: Prepared by authors with Eurostat data, "Use table at purchasers' prices" (domestic and imports)".

Table S10. Indirect requirements[a] on gross output (by products) per unit of additional final demand of health care services (SIOT)

Products	EU 28	EA 19	Belgium	Germany	Spain	France	Italy	Hungary	Austria	Greece	UK
**1**	0.0050	0.0045	0.0017	0.0034	0.0042	0.0036	0.0020	0.0038	0.0013	0.0031	0.0015
**2**	0.0028	0.0013	0.0002	0.0010	0.0013	0.0003	0.0009	0.0012	0.0010	0.0012	0.0039
**3**	0.0070	0.0063	0.0040	0.0034	0.0094	0.0037	0.0042	0.0030	0.0009	0.0011	0.0042
**4**	0.0013	0.0011	0.0007	0.0002	0.0015	0.0004	0.0015	0.0002	0.0001	0.0006	0.0004
**5**	0.0076	0.0063	0.0036	0.0043	0.0057	0.0023	0.0078	0.0024	0.0030	0.0019	0.0035
**6**	0.0039	0.0033	0.0035	0.0018	0.0014	0.0015	0.0041	0.0033	0.0008	0.0033	0.0009
**7**	0.0088	0.0075	0.0062	0.0013	0.0076	0.0015	0.0039	0.0015	0.0008	0.0008	0.0013
**8**	0.0035	0.0028	0.0074	0.0008	0.0015	0.0002	0.0020	0.0012	0.0016	0.0000	0.0014
**9**	0.0059	0.0050	0.0018	0.0037	0.0038	0.0019	0.0053	0.0017	0.0023	0.0034	0.0023
**10**	0.0082	0.0072	0.0017	0.0035	0.0057	0.0022	0.0078	0.0019	0.0017	0.0037	0.0022
**11**	0.0022	0.0015	0.0003	0.0009	0.0007	0.0003	0.0010	0.0002	0.0001	0.0000	0.0006
**12**	0.0019	0.0016	0.0003	0.0014	0.0013	0.0003	0.0016	0.0002	0.0004	0.0004	0.0008
**13**	0.0020	0.0018	0.0002	0.0007	0.0004	0.0004	0.0017	0.0001	0.0001	0.0001	0.0003
**14**	0.0024	0.0020	0.0002	0.0009	0.0004	0.0004	0.0024	0.0001	0.0004	0.0000	0.0007
**15**	0.0046	0.0040	0.0038	0.0018	0.0059	0.0018	0.0047	0.0023	0.0031	0.0006	0.0020
**16**	0.0122	0.0110	0.0042	0.0078	0.0170	0.0065	0.0119	0.0074	0.0169	0.0054	0.0108
**17**	0.0041	0.0037	0.0022	0.0023	0.0022	0.0027	0.0044	0.0021	0.0057	0.0012	0.0031
**18**	0.0091	0.0075	0.0066	0.0078	0.0068	0.0019	0.0096	0.0017	0.0101	0.0023	0.0086
**19**	0.0227	0.0195	0.0173	0.0132	0.0168	0.0076	0.0167	0.0093	0.0078	0.0127	0.0088
**20**	0.0201	0.0170	0.0118	0.0178	0.0171	0.0080	0.0153	0.0085	0.0072	0.0099	0.0117
**21**	0.0026	0.0024	0.0034	0.0002	0.0031	0.0021	0.0043	0.0009	0.0012	0.0014	0.0018
**22**	0.0036	0.0034	0.0022	0.0030	0.0022	0.0020	0.0033	0.0017	0.0025	0.0010	0.0007
**23**	0.0050	0.0047	0.0041	0.0038	0.0049	0.0036	0.0042	0.0018	0.0027	0.0027	0.0028
**24**	0.0065	0.0051	0.0095	0.0055	0.0013	0.0023	0.0065	0.0033	0.0038	0.0003	0.0064

Products	EU 28	EA 19	Belgium	Germany	Spain	France	Italy	Hungary	Austria	Greece	UK
**25**	0.0177	0.0168	0.0171	0.0125	0.0106	0.0147	0.0149	0.0105	0.0096	0.0091	0.0109
**26**	0.0110	0.0115	0.0101	0.0138	0.0116	0.0059	0.0094	0.0086	0.0088	0.0217	0.0028
**27**	0.0204	0.0186	0.0306	0.0132	0.0080	0.0132	0.0230	0.0091	0.0176	0.0080	0.0157
**28**	0.0007	0.0004	0.0000	0.0000	0.0000	0.0000	0.0011	0.0000	0.0000	0.0001	0.0024
**29**	0.0069	0.0056	0.0040	0.0031	0.0061	0.0023	0.0079	0.0029	0.0049	0.0029	0.0040
**30**	0.0187	0.0170	0.0233	0.0161	0.0134	0.0124	0.0146	0.0098	0.0109	0.0028	0.0159
**31**	0.0018	0.0014	0.0001	0.0039	0.0005	0.0000	0.0003	0.0016	0.0002	0.0000	0.0023
**32**	0.0014	0.0011	0.0007	0.0005	0.0012	0.0011	0.0008	0.0005	0.0003	0.0002	0.0019
**33**	0.0028	0.0023	0.0236	0.0014	0.0043	0.0003	0.0048	0.0028	0.0004	0.0001	0.0061
**34**	0.0005	0.0001	0.0000	0.0000	0.0000	0.0000	0.0017	0.0000	0.0000	0.0000	0.0060
**35**	0.0010	0.0009	0.0011	0.0007	0.0017	0.0003	0.0017	0.0002	0.0001	0.0005	0.0005
**36**	0.0022	0.0020	0.0043	0.0026	0.0013	0.0008	0.0015	0.0014	0.0009	0.0011	0.0015
**37**	0.0000	0.0000	0.0000	0.0000	0.0000	0.0000	0.0000	0.0000	0.0000	0.0000	0.0000
**TOTAL**	**0.2379**	**0.2084**	**0.2121**	**0.1582**	**0.1813**	**0.1082**	**0.2088**	**0.1070**	**0.1293**	**0.1038**	**0.1507**

[a] Indirect requirements have been calculated as the difference between the Leontief inverse matrix (symmetric input-output table) and the technical coefficients matrix (basic prices, domestic), also discounting the unit increase in production caused by the variation in final demand correspondent.

Source: Prepared by authors with Eurostat data, "Symmetric input-output table at basic prices (product by product) (domestic)".

Table S11. Direct requirements for GVA. 2010

	Health care activities. Direct requirements for GVA (euros of value added per unit of industry output) (use table)					Health care services. Direct requirements for GVA (euros of value added per unit of output, by products) (SIOT)				
	Health care activities	Average industries	Average industries (excl. ind. 37)	Average service industries	Average service industries (excl. ind. 37)	Health care services	Average products	Average products (excl. p. 37)	Average services	Average services (excl. p. 37)
EU (28)	0.644	0.468	0.454	0.588	0.565	0.644	0.467	0.452	0.582	0.560
EA (19)	0.667	0.473	0.458	0.600	0.577	0.667	0.471	0.456	0.594	0.571
Belgium	0.517	0.432	0.416	0.549	0.524	0.502	0.429	0.413	0.552	0.527
Denmark	0.673	0.483	0.469	0.576	0.552	:	:	:	:	:
Germany	:	:	:	:	:	0.678	0.482	0.468	0.604	0.582
Spain	0.662	0.473	0.458	0.618	0.596	0.662	0.460	0.445	0.620	0.599
France	0.756	0.474	0.459	0.617	0.595	0.757	0.477	0.462	0.618	0.597
Croatia	0.658	0.491	0.485	0.609	0.604	:	:	:	:	:
Italy	0.617	0.450	0.435	0.590	0.567	0.617	0.445	0.430	0.577	0.554
Cyprus	0.651	0.484	0.470	0.611	0.590	:	:	:	:	:
Hungary	0.561	0.467	0.452	0.600	0.578	0.556	0.461	0.446	0.596	0.574
Netherlands	0.679	0.472	0.458	0.587	0.564					
Austria	0.668	0.473	0.458	0.587	0.564	0.668	0.470	0.455	0.605	0.583
Poland	0.597	0.450	0.435	0.570	0.546	:	:	:	:	:
Portugal	0.578	0.454	0.439	0.587	0.564	:	:	:	:	:
Slovenia	0.633	0.465	0.450	0.589	0.566	:	:	:	:	:
Greece	:	:	:	:	:	0.730	0.506	0.492	0.616	0.594
UK	:	:	:	:	:	0.541	0.481	0.466	0.573	0.550

[a] (:) not available.

Source: Prepared by authors with Eurostat data, "Use table at basic prices (domestic)"/"Symmetric input-output table at basic prices (product by product) (domestic)"

Table S12. Direct and indirect (total) requirements for total GVA

	Direct and indirect (total) requirements for total GVA in all industries of the economy per unit of additional final demand (by product) (supply and use tables)					Direct and indirect (total) requirements for total GVA in whole economy per unit of additional final demand (by product) (SIOT)				
	**Health care services**	**Average products**	**Average products (excl. p. 37)**	**Average services**	**Average services (excl. p. 37)**	**Health care services**	**Average products**	**Average products (excl. p. 37)**	**Average services**	**Average services (excl. p. 37)**
EU (28)	0.899	0.858	0.854	0.912	0.907	0.899	0.858	0.854	0.912	0.907
EA (19)	0.901	0.836	0.831	0.903	0.898	0.901	0.836	0.831	0.903	0.898
Belgium	0.784	0.670	0.661	0.788	0.776	0.781	0.667	0.657	0.798	0.786
Denmark	0.829	0.739	0.734	0.801	0.796	:	:	:	:	:
Germany	:	:	:	:	:	0.887	0.793	0.787	0.894	0.888
Spain	0.879	0.789	0.783	0.888	0.882	0.879	0.785	0.779	0.898	0.892
France	0.890	0.771	0.765	0.880	0.874	0.891	0.771	0.765	0.880	0.873
Croatia	0.857	0.732	0.726	0.819	0.812	:	:	:	:	:
Italy	0.859	0.769	0.763	0.875	0.869	0.859	0.769	0.762	0.876	0.869
Cyprus	0.800	0.715	0.707	0.792	0.781	:	:	:	:	:
Hungary	0.735	0.644	0.634	0.782	0.770	0.730	0.656	0.647	0.801	0.790
Netherlands	0.851	0.740	0.733	0.836	0.827	:	:	:	:	:
Austria	0.833	0.731	0.724	0.837	0.828	0.833	0.729	0.721	0.858	0.850
Poland	0.825	0.743	0.735	0.834	0.825	:	:	:	:	:
Portugal	0.796	0.728	0.720	0.849	0.840	:	:	:	:	:
Slovenia	0.780	0.707	0.699	0.807	0.796	:	:	:	:	:
Greece	:	:	:	:	:	0.893	0.797	0.791	0.881	0.874
UK	:	:	:	:	:	0.751	0.757	0.750	0.835	0.826

[a] (:) not available.

Source: Prepared by authors with Eurostat data, "Use table at basic prices (domestic)”/"Supply table at basic prices"/”Symmetric input-output table at basic prices (product by product) (domestic)".

Table S13. Direct requirements for hours worked[a]

	Health care activities. Direct requirements for hours worked (number of hours per thousand EUR of industry output) (use table)			Health care services. Direct requirements for hours worked (number of hours per thousand EUR of output, by products) (SIOT)				
	**Health care activities**	**Average industries**	**Average service industries**	**Health care services**	**Average products**	**Average products (excl. p. 37)**	**Average services**	**Average services (excl. p. 37)**
EU (28)	22.253	17.313	21.985	22.683	17.185	15.209	21.305	17.583
EA (19)	19.968	15.385	21.321	20.387	15.302	12.840	20.829	16.210
Belgium	13.480	12.299	18.396	14.405	11.945	8.665	17.750	11.512
Denmark	16.538	10.177	14.052	:	:	:	:	:
Germany	:	:	:	21.801	13.855	12.311	18.735	15.918
Spain	19.472	17.463	24.217	19.769	17.274	15.454	23.109	19.793
France	19.170	13.080	18.725	19.368	13.176	10.806	18.884	14.462
Croatia	69.594	46.037	49.496	:	:	:	:	:
Italy	17.437	16.174	21.975	17.814	16.044	13.035	21.424	15.705
Cyprus	24.569	31.315	37.583	:	:	:	:	:
Hungary	65.540	56.493	79.777	65.572	55.152	38.176	77.818	45.124
Netherlands	16.871	11.307	16.170	:	:	:	:	:
Austria	19.752	14.596	19.694	20.493	14.480	12.782	18.514	15.342
Poland	74.160	46.224	53.686	:	:	:	:	:
Portugal	32.189	30.033	38.361	:	:	:	:	:
Slovenia	32.473	26.607	31.446	:	:	:	:	:
Greece	:	:	:	25.088	30.051	27.456	32.322	27.258
UK	:	:	:	19.894	13.819	13.917	16.406	16.747

[a] hours worked by industry (not by product); [b] (:) not available.

Source: Prepared by authors with Eurostat data, "Use table at basic prices (domestic)"/"Symmetric input-output table at basic prices (product by product) (domestic)"/"National Accounts employment data by industry".

Table S14. Direct and indirect (total) requirements for total hours worked[a]

	Direct and indirect (total) requirements for total hours worked in all industries of economy per unit of additional final demand (by product) (supply and use tables)					Direct and indirect (total) requirements for total hours worked in whole economy per unit of additional final demand (by product) (SIOT)				
	**Health care services**	**Average products**	**Average products (excl. p. 37)**	**Average services**	**Average services (excl. p. 37)**	**Health care services**	**Average products**	**Average products (excl. p. 37)**	**Average services**	**Average services (excl. p. 37)**
EU (28)	30.375	30.087	28.452	31.940	28.773	30.741	29.930	28.303	31.255	28.075
EA (19)	26.454	25.318	23.134	29.216	25.064	26.815	25.146	22.957	28.702	24.521
Belgium	19.451	17.505	14.380	23.459	17.539	20.742	16.941	13.800	22.776	16.818
Denmark	19.443	13.792	13.352	16.421	15.687	:	:	:	:	:
Germany	:	:	:	:	:	26.997	21.176	19.836	25.204	22.747
Spain	27.195	28.012	26.490	32.781	30.002	27.403	28.005	26.483	31.514	28.665
France	22.066	19.682	17.494	24.163	20.034	22.169	19.490	17.296	24.115	19.984
Croatia	84.640	61.389	61.024	63.101	62.465	:	:	:	:	:
Italy	24.880	25.926	23.191	30.306	25.080	25.128	25.626	22.883	29.771	24.516
Cyprus	32.205	40.857	34.932	44.026	32.352	:	:	:	:	:
Hungary	78.881	69.841	53.272	93.154	61.312	78.772	69.664	53.090	91.273	59.326
Netherlands	21.046	16.973	16.058	21.330	19.741	:	:	:	:	:
Austria	23.678	21.363	19.856	25.328	22.535	24.291	21.463	19.959	24.265	21.412
Poland	92.586	72.978	73.279	75.727	76.483	:	:	:	:	:
Portugal	44.680	46.167	43.037	52.835	46.946	:	:	:	:	:
Slovenia	40.160	39.216	37.725	41.880	39.046	:	:	:	:	:
Greece	:	:	:	:	:	33.992	43.307	41.081	41.607	37.059
UK	:	:	:	:	:	26.504	22.437	22.775	24.827	25.635

[a] hours worked by industry (not by product); [b] (:) not available.

Source: Prepared by authors with Eurostat data. "Use table at basic prices (domestic)”/"Supply table at basic prices"/”Symmetric input-output table at basic prices (product by product) (domestic)"/“National Accounts employment data by industry”.

Table S15. Final consumption expenditure per capita on health care services (use table. basic prices. domestic and imports). Index EU28 = 100. 2010

	**Final consumption expenditure per capita on health care services (use table. basic prices. domestic and imports). Index EU28 = 100. 2010**				
	**HH FCE per capita (in PPS_EU28)**	**GG FCE per capita (in PPS-EU28)**		**HH FCE per capita (in PPS_EU28)**	**GG FCE per capita (in PPS-EU28)**
EU-28	100.00	100.00	Latvia	82.24	36.24
EA-19	115.44	97.59	Lithuania	25.56	53.12
Belgium	133.02	105.23	Luxembourg	30.11	84.18
Bulgaria	60.48	31.39	Hungary	88.45	58.30
Czech Rep.	52.97	99.41	Malta	71.92	63.91
Denmark	72.81	130.71	Netherlands	81.82	101.63
Germany	166.44	112.75	Austria	104.48	102.59
Estonia	41.69	57.89	Poland	70.00	51.58
Ireland	119.25	110.27	Portugal	119.96	60.73
Greece	165.65	54.44	Romania	74.06	62.82
Spain	90.96	73.60	Slovenia	71.89	72.75
France	113.49	107.60	Slovakia	81.20	65.81
Croatia	65.70	48.50	Finland	123.34	110.64
Italy	83.51	103.13	Sweden	71.30	112.30
Cyprus	167.53	42.73	UK	70.47	152.88

Source: Prepared by authors with Eurostat data. "Use table at basic prices (domestic and imports)"/ "Purchasing power parities (PPPs)"/ "Euro/ECU exchange rates - annual data"/ "Population on 1 January by age and sex".

FIGURE S1 Comparison of Apparent labour productivity (ALP-output) on health care activities (in PPS_EU28 per hours worked) (Index EU28=100), using PPPs for GDP and for health. 2010

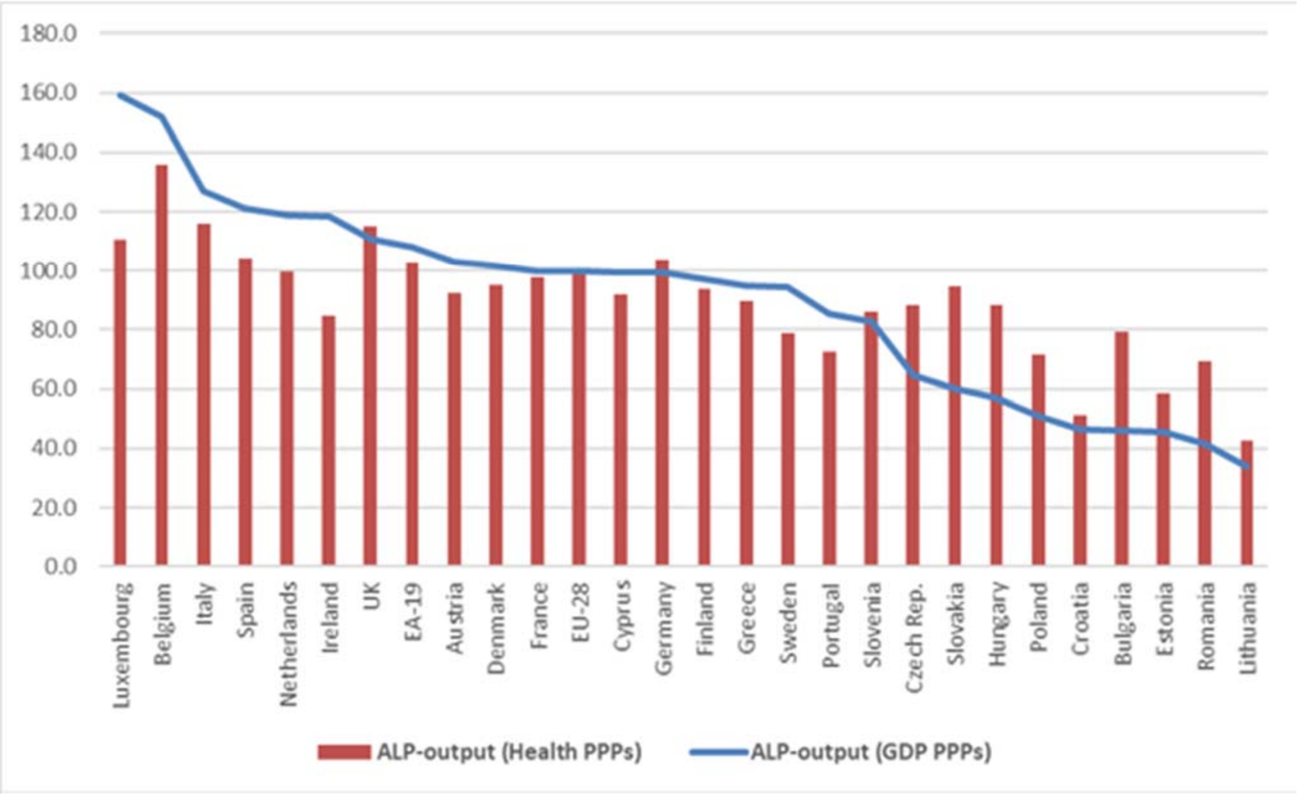

Source: Prepared by authors with Eurostat data, "Supply table at basic prices"/"Use table at basic prices (domestic)"/"National Accounts employment data by industry"/"Purchasing power parities (PPPs)"/"Euro/ECU exchange rates - annual data".
